# Hijacking of internal calcium dynamics by intracellularly residing viral rhodopsins

**DOI:** 10.1038/s41467-023-44548-6

**Published:** 2024-01-02

**Authors:** Ana-Sofia Eria-Oliveira, Mathilde Folacci, Anne Amandine Chassot, Sandrine Fedou, Nadine Thézé, Dmitrii Zabelskii, Alexey Alekseev, Ernst Bamberg, Valentin Gordeliy, Guillaume Sandoz, Michel Vivaudou

**Affiliations:** 1 Univ. Grenoble Alpes, CEA, CNRS, IBS, Grenoble, France; 2 Université Côte d’Azur, CNRS, INSERM, iBV, Nice, France; 3 Laboratories of Excellence, Ion Channel Science and Therapeutics, Nice, France; 4 Fédération Hospitalo-Universitaire InovPain, Cote d’Azur University, University Hospital Center Nice, Nice, France; 5 Univ. Bordeaux, Inserm, BRIC, UMR, 1312 Bordeaux, France; 6 European XFEL, Schenefeld, Germany; 7 Advanced Optogenes Group, Institute for Auditory Neuroscience and InnerEarLab, University Medical Center Göttingen, Göttingen, Germany; 8 Cluster of Excellence “Multiscale Bioimaging: from Molecular Machines to Networks of Excitable Cells” (MBExC), University of Göttingen, Göttingen, Germany; 9 Max Planck Institute of Biophysics, Frankfurt am Main, Germany; 10 Institute of Biological Information Processing (IBI-7: Structural Biochemistry), Forschungszentrum Jülich, Jülich, Germany; 11 Present Address: Department of Biomedicine, Aarhus University, Aarhus, Denmark

**Keywords:** Calcium signalling, Optogenetics, Virology, Membrane proteins

## Abstract

Rhodopsins are ubiquitous light-driven membrane proteins with diverse functions, including ion transport. Widely distributed, they are also coded in the genomes of giant viruses infecting phytoplankton where their function is not settled. Here, we examine the properties of OLPVR1 (Organic Lake Phycodnavirus Rhodopsin) and two other type 1 viral channelrhodopsins (VCR1s), and demonstrate that VCR1s accumulate exclusively intracellularly, and, upon illumination, induce calcium release from intracellular IP_3_-dependent stores. In vivo, this light-induced calcium release is sufficient to remote control muscle contraction in VCR1-expressing tadpoles. VCR1s natively confer light-induced Ca^2+^ release, suggesting a distinct mechanism for reshaping the response to light of virus-infected algae. The ability of VCR1s to photorelease calcium without altering plasma membrane electrical properties marks them as potential precursors for optogenetics tools, with potential applications in basic research and medicine.

## Abstract (web summary)

Rhodopsins are ubiquitous light-driven membrane proteins that have diverse functions in nature, and value as optogenetics tools. Here the authors characterise type 1 viral channelrhodopsins, showing that they regulate intracellular calcium and can be used for the photocontrol of muscle contraction in vivo.

## Introduction

Rhodopsins are ubiquitous integral membrane proteins found in many living organisms, from bacteria to man^1^. Although their functions are diverse, they share the property of being driven by light, due to the presence within their structure of the light-isomerizable retinal^2^. Many microbial rhodopsins are ion transporters, either photon-driven ion pumps like bacteriorhodopsins, or photon-gated ion channels like channelrhodopsins. When exogenously expressed in the plasma membrane of mammalian cells, channelrhodopsins provide a means to modify cell excitability with high spatiotemporal resolution upon illumination, a property at the origin of optogenetics.

Rhodopsin genes have been identified in the genomes of giant viruses^3–5^. Metagenomic and phylogenetic sequence analysis shows that viral rhodopsins (VRs) are extremely abundant in marine environments. They form a monophyletic group of proteins within the rhodopsin superfamily that splits into two distinct branches: VRs of type 1 and type 2^3,6–8^. VRs have the expected 7-transmembrane-helices topology of rhodopsins, but show only distant sequence similarity to microbial rhodopsins of known functions such as channelrhodopsin-2 (ChR2). The few VRs that have been functionally characterized in heterologous systems encode light-driven cation channels (Type 1 VirChR1 and VirRDTS) with weak proton-pumping activity^4,8^. These channelrhodopsins are thought to reside in the plasma membrane of the virus-infected phytoplankton host and to enhance phototaxis through a process linking plasma membrane ion flux to intracellular calcium which drives flagellar motion^9–11^.

Here, we study OLPVR1 (Organic Lake phycodnavirus rhodopsin). In *Xenopus* oocytes and a mammalian cell line, we demonstrate that native OLPVR1 strictly expresses intracellularly, localizes to the endoplasmic reticulum, and triggers an increase in cytoplasmic calcium proportional to the light power applied. Such function, so far unique among rhodopsins, uncovers an unexpected facet of the interactions of giant viruses with their phytoplankton hosts. It also suggests optogenetic use of VCR1s in a variety of cells where a straight link between intracellular calcium and cell function exists. As proof-of-concept of such use, we show here that light irradiation reversibly modified tail movements of OLPVR1-expressing frog tadpoles.

## Results

### OLPVR1 expression in oocytes elicits light-induced Ca^2+^-activated chloride currents

**Fig. 1:**
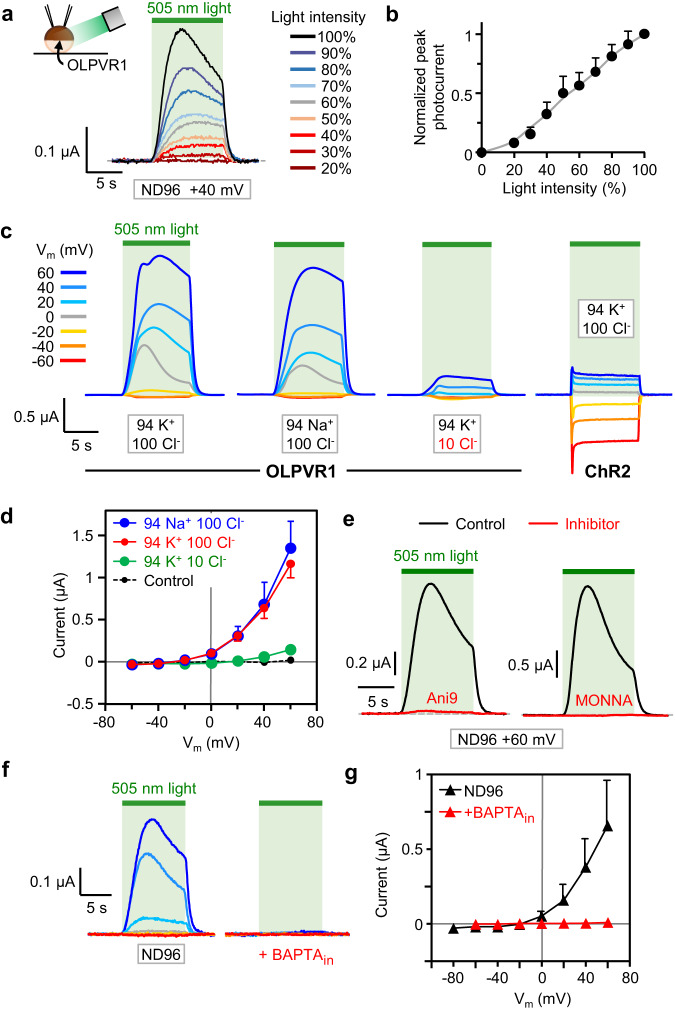
Photoactivation of OLPVR1 elicits CaCC currents in *Xenopus* oocytes. **a** Responses to a 10-s pulse of light of decreasing intensity. Current records were taken every 50 s from the same oocyte injected with 30 ng OLPVR1 RNA clamped at +40 mV. Bath solution was ND96. **b** Average peak current, normalized to current at 100% intensity (75 µW mm^-2^), vs. light intensity obtained with the protocol of **a**, applied to 4 oocytes (Error bars, SEM). **c** Photocurrents at different holding voltages from oocytes expressing OLPVR1 (30 ng RNA) or ChR2 (7.5 ng), in the specified different bath solutions. **d** OLPVR1 current-voltage relationships obtained from records as in **c**, in different extracellular ionic conditions. Control refers to currents recorded in non-injected oocytes. Currents were measured after 10-s illumination. (Error bars, SEM; n = 15, 11, 9, and 8 for 94 K^+^ 100 Cl^-^, 94 Na^+^ 100 Cl^-^, 94 K^+^ 10 Cl^-^, and control, respectively). **e** OLPVR1 photocurrents recorded before (Black) and after (Red) 60’ incubation in ND96 solution containing 30 µM Ani9 or MONNA, inhibitors of TMEM16A CaCCs. Statistics are shown in Supplementary Fig. 1. **f** Photocurrents from OLPVR1-expressing oocytes (7.5 ng RNA) with and without intracellular injection of BAPTA (BAPTA_in_) in ND96 bath solution. **g** Average peak photocurrent vs voltage in ND96 solution with and without injected BAPTA. (Error bars, SEM; n = 9 for ND96, n = 3 for +BAPTA_in_). Source data are provided as a Source Data file.

Whereas naïve oocytes produced no light-sensitive currents (Fig. 1d), illumination of oocytes expressing OLPVR1 induced a current which reached a peak within seconds, slowly desensitized during illumination, and rapidly disappeared in the dark. The amplitude of photocurrents correlated with the light intensity (Fig. 1a, b). The selectivity of OLPVR1 photocurrents was investigated by changing the ion composition of the bathing solution (Fig. 1c, d). Exchanging K^+^ for Na^+^ did not affect the amplitude of the currents. Lowering external Cl^-^ from 100 to 10 mM caused a drastic reduction in current amplitude while shifting the reversal potential from ~−25 to ~+10 mV (Fig. 1d). The chloride selectivity of OLPVR1 photocurrents and their strong outward rectification are properties of the endogenous Ca^2+^-activated Cl^-^ currents (CaCCs) of *Xenopus* oocytes, known to be carried by TMEM16A channels^12,13^. We, therefore, tested two TMEM16A inhibitors, Ani9^14^ and MONNA^15^. Ani9 and MONNA, at a concentration of 30 µM in ND96 solution, blocked photocurrents by 93.4 ± 1.4% and 98.9 ± 0.4%, respectively (Fig. 1e). Full 100% inhibition was not achieved, more noticeably with Ani9, but this is more likely due to incomplete block of CaCCs by these agents. Indeed, similar incomplete inhibition was obtained when Ani9 and MONNA were tested on CaCCs induced by the release of internal Ca^2+^ subsequent to the activation of Gq-coupled M3 receptors (Supplementary Fig. 1)^16^.

Therefore, OLPVR1 expression produces light-activated currents with the features of the endogenous CaCCs of oocytes.

### OLPVR1-mediated photocurrents require intracellular calcium

In order to understand the link between OLPVR1 and CaCCs, we recorded photocurrents before and after microinjection of the fast Ca^2+^ chelator BAPTA in oocytes. Such treatment proved sufficient to eliminate all currents elicited by the activation of M3 receptors (Supplementary Fig. 1). BAPTA injection in OLPVR1-expressing oocytes also eliminated all light-activated currents (Fig. 1f, g), demonstrating that photocurrents are mediated by changes in intracellular calcium. Whether oocytes were bathed in normal 1.8 mM extracellular Ca^2+^ (Fig. 1f, g), or elevated 49 mM Ca^2+^ (Supplementary Fig. 2b, d), no photocurrent was detectable after BAPTA injection, given that the signal/noise ratio of our recordings afforded a resolution >~1 nA. In particular, no inward current at negative potentials was observed, ruling out an influx of external Ca^2+^, directly or indirectly linked to OLPVR1.

We tested channelrhodopsin-2 (ChR2) as a control in the same conditions. As previously documented^17^, the profile and BAPTA_in_ modulation of ChR2 photocurrents were completely different than those of OLPVR1 (Supplementary Fig. S2). In normal external Ca^2+^ (Supplementary Fig. 2e, g), ChR2 cationic photocurrents were little affected by BAPTA_in_. In elevated external Ca^2+^ (Supplementary Fig. 2f, h), ChR2, which is permeable to Ca^2+^ ions, produced currents different than in low Ca^2+^, especially at the most negative potentials where Ca^2+^ entry was large enough to activate CaCC currents^17^. In agreement with this mechanism, BAPTA_in_ removed these large CaCC currents without affecting the intrinsic ChR2 currents.

Another remarkable difference between ChR2 and OLPVR1 is the significantly slower kinetics of the response to light of OLPVR1. The half-times of activation are ~2 s for OLPVR1 and <0.1 s for ChR2 (Supplementary Fig. 3). Fast activation of ChR2 photocurrents reflects the known intrinsic capabilities of ChR2 to sense light and conduct ions. Slow activation of OLPVR1 photocurrents is compatible with an action of OLPVR1 mediated by a diffusion-limited Ca^2+^-dependent process akin to the action of Gq-coupled M3 receptors (Supplementary Fig. 3). In fact, CaCC response after OLPVR1 illumination was twice as slow as that after M3 activation by ACh.

These experiments show that, although both OLPVR1 and ChR2 can cause opening of CaCC channels, the mechanisms are fundamentally different. OLPVR1-induced photocurrents are entirely carried by CaCC channels activated by an elevation of intracellular Ca^2+^ independent of external Ca^2+^. ChR2 photocurrents result from cations flowing through the protein and, in conditions favoring Ca^2+^ permeation, an additional contribution from Ca^2+^-activated currents.

### Intracellular located OLPVR1 elicits release of calcium from IP_3_-dependent stores

**Fig. 2:**
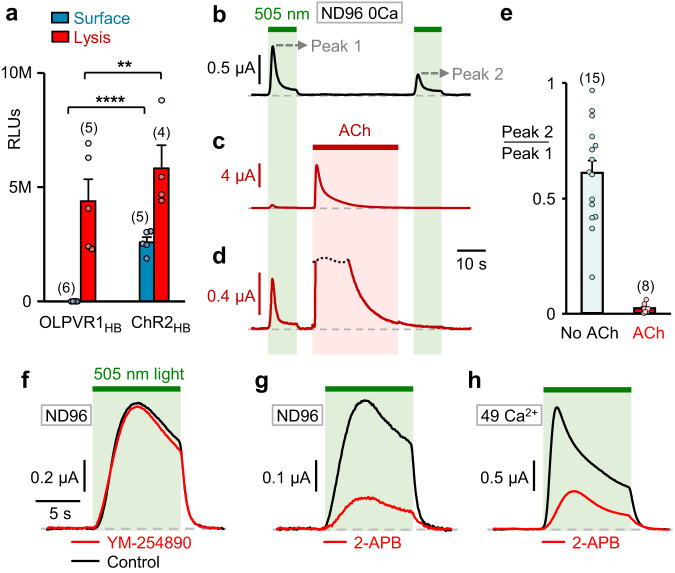
OLPVR1 is expressed intracellularly and activates surface CaCCs through release of intracellular Ca^2+^. **a** Surface expression of HiBit-tagged OLPVR1_HB_ compared to ChR2_HB_ measured using XenoGlo technique. OLPVR1, in contrast with ChR2, is not expressed at the surface membrane of oocytes. Mean luminescence recorded in oocytes injected with 7.5 ng RNA coding for OLPVR1_HB_ and ChR2_HB_ before (blue) and after (red) membrane permeabilization. The OLPVR1 surface value is 2360 ± 700 RLUs. The numbers of oocytes batches are in parentheses (Error bars, SEM). **P = 0.007, ****P < 0.0001 two-way ANOVA with Sidak’s post hoc test (DF = 60). **b** Photocurrents elicited by successive 10-s illuminations separated by a 40-s dark interval. Oocytes coexpressing OLPVR1 (30 ng RNA) and Gq-coupled muscarinic M3 receptor (2.5 ng) were bathed in ND96 0Ca solution. **c, d** Between the 2 illuminations, activation of M3 by ACh (5 µM; 30 s) induced Ca^2+^ release and large transient CaCC currents. Panel d is an enlarged version of c, showing a drastic reduction of the second photocurrent. **e** Average ratios of OLPVR1 peak current induced by the second illumination (Peak 2) over that of the first (Peak 1) with and without ACh application in between. Numbers of oocytes are in parentheses (Error bars, SEM). **f** Photocurrents at +60 mV in ND96 solution from oocytes expressing OLPVR1 (7.5 ng RNA) before (Control) and after 10’ incubation with 10 µM YM-254890. **g, h** Representative photocurrents at +60 mV in ND96 (g) or 49 Ca^2+^(h) solution from oocytes expressing OLPVR1 (7.5 ng RNA) before (Control) and after 60’ incubation with 100 µM 2-APB. Statistics are shown in Supplementary Fig. 1. Source data are provided as a Source Data file.

Analysis of the atomic structure of OLPVR1 and its high phylogenetic similarity to VirChR1 (61% sequence identity – See Supplementary Note 1) suggested that OLPVR1 could function like VirChR1 as a cation-conducting channel^8^. The distinct effects of OLPVR1 in oocytes proved that it is expressed and functional. Our observations did not, however, show evidence of plasma membrane currents carried by OLPVR1 itself. This could be because OLPVR1 is not at the plasma membrane and we can only record the activity of channels present at the plasma membrane. To address the localization of OLPVR1, we used the XenoGlo technique, an adaptation of the Nano-Glo® system (Promega) to *Xenopus* oocytes. The HiBiT tag was inserted at the N-terminal extracellular end of OLPVR1 and ChR2, to produce functional OLPVR1_HB_ and ChR2_HB_ (Supplementary Fig. 4). The data (Fig. 2a) show that OLPVR1 was not detected at the oocyte surface in contrast to ChR2 which was highly expressed. Nonetheless, once oocytes were permeabilized, OLPVR1 was found in large amounts, comparable to the total amount of ChR2. This implies that OLPVR1 is not addressed to the surface membrane but is abundant in intracellular membranes.

As shown above, the intermediary between OLPVR1 photoactivation and CaCCs is intracellular Ca^2+^. Could OLPVR1 modulate release of Ca^2+^ from intracellular stores? And, if so, which stores? These questions were addressed by observing the impact of Ca^2+^- stores depletion on OLPVR1 photocurrents. We coexpressed OLPVR1 and M3. Activation of M3 by its agonist Acetylcholine triggers the Gq-signaling cascade, the production of IP_3_, the release of Ca^2+^ stored in the endoplasmic reticulum (ER) by IP_3_ receptors, and the opening of CaCC channels^16^. Under prolonged stimulation with a saturating ACh concentration (5 µM), in absence of extracellular Ca^2+^, the signature CaCCs peaked within 2 s (Fig. 2c) and decayed by >99% within 30 s, an indication that Ca^2+^ stores have been emptied. We measured OLPVR1 photocurrents before and after such ACh application (Fig. 2c-e) and observed that photocurrents were barely detectable after ACh application, their amplitude decreasing to 2 ± 0.6% of the initial amplitude. In control conditions, the same protocol without intervening ACh application showed a decrease of photocurrents of 39 ± 6% (Fig. 2b, e).

These experiments suggest that the Ca^2+^ ions released by OLPVR1 come from the same stores as those released by IP_3_ receptors, presumably the endoplasmic reticulum. We also note that M3 stimulation produced ~20-fold larger and ~2-fold faster currents than OLPVR1. Such difference is not unexpected considering the different natures of the stimuli. Bath-applied ACh reaches the entire population of surface M3 receptors and the produced IP_3_ molecules can diffuse to reach most intracellular IP_3_ receptors. In contrast, light can only penetrate a few microns below the surface of opaque oocytes and reaches a small fraction ( < 5%) of the OLPVR1 proteins present (See Methods). As such, the extent of Ca^2+^ released from the ER upon M3 activation is much higher and the Ca^2+^ concentration near the surface membrane where CaCCs reside reaches the CaCC activation threshold faster.

### OLPVR1 integrates tightly with the calcium-release mechanism of *Xenopus* oocytes

To further examine the link between Ca^2+^ release and OLPVR1, we utilized 2 inhibitors: YM-254890 targets Gq proteins^18^, and 2-aminoethoxydiphenyl borate (2-APB) targets both IP_3_ receptors and Store-Operated Calcium Entry (SOCE)^19^. Their effects on OLPVR1 photocurrents were tested at saturating concentrations that blocked >98% of the response to M3 activation in oocytes (Fig. 2f-h*&* Supplementary Fig. 1*)*. The Gq inhibitor YM−254890 did not affect the OLPVR1 photocurrents, confirming that OLPVR1 mobilizes calcium release independently of the Gq pathway. 2-APB did not have such a clear-cut effect. It decreased OLPVR1 currents considerably but far from completely. On average, 2-APB reduced photocurrents amplitude by 75%. This suggests that the OLPVR1 photocurrents are largely contributed by the action of IP_3_R and/or SOCE, likely as a consequence of the positive feedback amplification of calcium signaling. This observation applies with the caveat that we do not know the direct effects of 2-APB on OLPVR1, which might confound our conclusions.

The fact that 2-APB fully blocks IP_3_-dependent currents elicited by M3 activation, but only partially blocks OLPVR1-induced photocurrents, suggests that OLPVR1 can induce a release of Ca^2+^ from stores even when IP_3_ receptors are inoperative. Thus, OLPVR1 can act independently of IP_3_ receptors although it appears that IP_3_ receptors contribute a large fraction of the OLPVR1 photocurrents – the 2-APB-sensitive fraction – likely because the Ca^2+^ release initiated by the fraction of illuminated OLPVR1s is propagated through opening of Ca^2+^-activated IP_3_ receptors^20,21^.

### ER-localized OLPVR1 increases intracellular calcium in HEK293T cells

To generalize our results, we turned to a mammalian cell line, HEK293T cells, and recorded currents with the patch clamp technique in the whole-cell configuration. In that configuration, the cytoplasm is perfused with the pipette solution. Unless otherwise specified, we added 10 µM of the slow chelator EGTA, a quantity sufficient to chelate the contaminant Ca^2+^ in our pipette solutions, but low enough to preserve Ca^2+^ signals as demonstrated below.

**Fig. 3:**
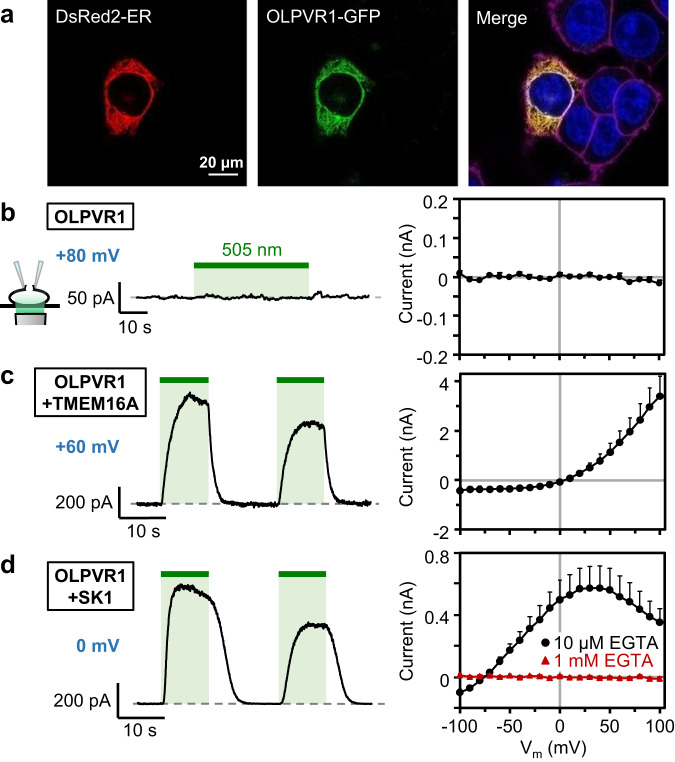
In mammalian cells, OLPVR1 localizes to the ER and activates surface Ca^2+^-activated channels through release of intracellular Ca^2+^. **a** Confocal images of HEK293T cells cotransfected with plasmids for expression of the ER-marker DsRed2-ER (red) and GFP-fused OLPVR1 (green). Merge panel shows in yellow, overlapping ER (red) and OLPVR1 (green) signals. The nucleus is in blue and the plasma membrane in magenta. Image is representative of 18 cells from 3 independent transfections. **b-d** Whole-cell recordings were obtained from HEK293T cells transfected with OLPVR1 alone (**b**; n = 7) or with OLPVR1 and, either TMEM16A (**c**; n = 5) or SK1 (**d**; n = 8). Left panels: Current responses to illumination (green bars, 505 nm light). Cells were held at the indicated voltages. Right panels: Light-induced current (peak current elicited by first illumination – current before illumination) vs. voltage (Error bars, SEM). Currents were elicited by 400-ms voltage ramps from −100 to +100 mV repeated every second. The pipette solution had 10 µM EGTA, except for the current-voltage curve drawn in red (right panel of **d**) where it had 1 mM EGTA (n = 7). Source data are provided as a Source Data file.

Initial experiments showed that light induced no detectable current in HEK293T cells transfected with OLPVR1 (Fig. 3b & Supplementary Fig. 5). Accordingly, confocal fluorescence imaging showed that OLPVR1 was not expressed at the plasma membrane but co-localized with an ER marker (Fig. 3a). Unlike *Xenopus* oocytes, HEK293T cells lack endogenous Ca^2+^-sensitive channels to serve as electrophysiological readouts of intracellular Ca^2+^. To match oocyte conditions, HEK293T cells were transfected with OLPVR1 and TMEM16A (aka ANO1) – a gene coding for the same Ca^2+^-activated chloride channels as the CaCCs of *Xenopus* oocytes^12^. As shown in Fig. 3c, light pulses transiently induced outwardly rectifying currents reversing at ~0 mV, the expected chloride Nernst potential. Similar results were obtained with TMEM16B/ANO2, a homologue of TMEM16A found in neuronal cells^22^ (Supplementary Fig. 5).

Further tests were performed with a different Ca^2+^ sensor, the small-conductance Ca^2+^-activated potassium channel SK1^23^. Cells transfected with OLPVR1 and SK1 displayed large photocurrents that reversed near −80 mV, the K^+^ Nernst potential, and had a typical SK1 current-voltage relationship (Fig. 3d). The photocurrents disappeared when the slow chelator EGTA was included in the pipette solution at 1 mM, ruling out the type of tight association between the OLPVR1-dependent Ca^2+^ source and surface channels that has been found between IP_3_R and CaCCs^24^.

### OLPVR1 activates a fluorescence Ca^2+^ sensor in its immediate vicinity even in the presence of the fast Ca^2+^chelator BAPTA-AM

**Fig. 4:**
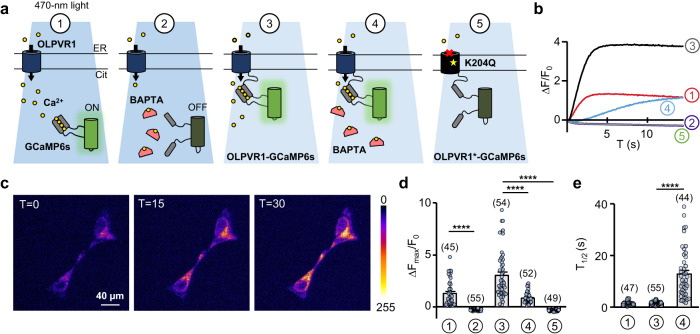
A fused Calcium sensor shows OLPVR1-delimited Ca^2+^ influx from the ER. **a** Schematic representation of the calcium-imaging conditions tested. HEK293T cells were transfected with plasmids encoding for wild-type OLPVR1 and GCaMP6s (1&2), the fusion construct OLPVR1-GCaMP6s (3&4) or loss-of-function OLPVR1(K204Q)-GCaMP6s (5) and imaged with an epifluorescence microscope under 470-nm light in the absence (1,3&5) or presence (2&5) of 15 µM BAPTA-AM. **b** Time courses of fluorescence changes upon light application in the conditions described in a. **c** Confocal images of cells transfected with a plasmid coding for OLPVR1-GCaMP6s after 2 h incubation in 15 µM BAPTA-AM at T = 0, 15 and 30 s after light application. T = 0 is the first frame acquired after light is turned on. **d** Average maximal fluorescence changes upon light application of cells from at least 3 independent transfections in the conditions shown in a. Numbers of cells are in parentheses. (Error bars, SEM) **e** Half times of rise in fluorescence during illumination for conditions 1, 3 and 4. Numbers of cells are in parentheses (Error bars, SEM). **** p < 0.0001, one-way ANOVA, Tukey’s multiple comparisons test, *F*_*D*_ (DFn 4, _*D*_*F*d 250) = 71.2; *F*_*E*_ (DFn 2, D*F*d 143) = 68.8. Source data are provided as a Source Data file.

We followed multiple strategies to boost OLPVR1 plasma membrane expression, but this was insufficient to measure ionic flux through OLPVR1 (Supplementary Fig. 6 & Supplementary Note 2). As an alternate approach to assess Ca^2+^ transport through OLPVR1, we fused OLPVR1 to the genetically-encoded fluorescent Ca^2+^ probe GCaMP6s^25,26^. OLPVR1-GCaMP6s proteins remained localized to the ER in HEK293T cells (Supplementary Fig. 7). The fluorescence signal from GCaMP6s, either coexpressed with, or fused to, OLPVR1, reported the expected (Supplementary Fig. 3) OLPVR1-triggered cytosolic Ca^2+^ increase with a half time of ~2 s (Fig. 4, conditions 1&3).

Incubating the cells with 15 µM BAPTA-AM - a membrane-permeant inactive BAPTA derivative that is converted to the membrane-impermeant active BAPTA in the cell cytoplasm - for 2 h was sufficient to chelate bulk cytosolic Ca^2+^ as reported by the absence of signal from free cytosolic GCaMP6s (Fig. 4, conditions 1&2). BAPTA-AM, however, did not eliminate the fluorescence signal in OLPVR1-GCaMP6s-expressing cells although it significantly reduced amplitude ( ~ 2X) and increased rise time ( ~ 8X) (Fig. 4, condition 4).

Thus, in absence of free Ca^2+^ ions in the cytoplasm with BAPTA, the cytosolic Ca^2+^ sensor tethered to OLPVR1 still detects Ca^2+^ ions upon OLPVR1 activation. These ions must therefore come from the ER and reach the sensor faster than the chelator BAPTA, implying nm proximity between ions entry point and sensor^27^. A direct permeation through OLPVR1 or adjacent Ca^2+^ channels is required.

To further detail the mechanism, we used the OLPVR1 mutant K204Q, where the replacement of the conserved retinal-binding lysine by a glutamine is predicted to disrupt function with minimal structural impact^8^. The mutant fusion OLPVR1(K204Q)-GCaMP6s had the same expression pattern as the wild-type (Supplementary Fig. 7), but produced no Ca^2+^ signal upon illumination (Fig. 4, condition 5). We then coexpressed OLPVR1(K204Q)-GCaMP6s with active wild-type OLPVR1, expecting close proximity between the 2 proteins because they both reside at the ER and are predicted to associate as dimers^8^. In control condition, the sensor tethered to the inactive mutant detected the global Ca^2+^ release induced by wild-type OLPVR1. With BAPTA, the sensor did not detect the OLPVR1-associated local Ca^2+^ entry in spite of their close proximity (Supplementary Fig. 8). Because BAPTA is able to intercept Ca^2+^ ions when their release site and the Ca^2+^ sensor are on separate proteins, its lack of effect on OLPVR1-GCaMP6s suggests that OLPVR1 mediates Ca^2+^ entry.

These results are in agreement with the experiments in Fig. 2g&h, which already implied two ER Ca^2+^ release pathways upon OLPVR1 activation, direct light-activated permeation through OLPVR1 and subsequent Ca^2+^-induced Ca^2+^ release through IP_3_ receptors.

### Light-driven muscle contraction endowed by OLPVR1

**Fig. 5:**
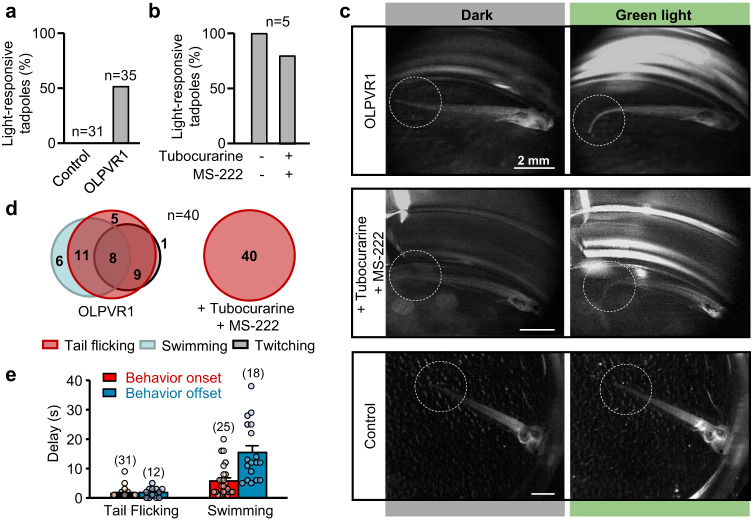
Green light induces motion of *Xenopus* tadpoles expressing OLPVR1. **a** Tadpoles from 3 independent batches of mRNA-injected embryos (Control injected with 250 pg lacZ mRNA, and OLPVR1 injected with 1 ng OLPVR1 mRNA) were subjected to 2-200-s green-light pulses and observed for light-triggered behaviour. No lacZ tadpole (n = 31) demonstrated green-light response. 51.4% of OLPVR1-injected tadpoles (n = 35) displayed green-light-dependent motion. **b** OLPVR1-injected light-responsive tadpoles from 1 batch (n = 5) were subjected to 0.5 mM of tubocurarine and 0.02% MS-222. 80% of tadpoles retained light-responsiveness as seen in middle panel of c. **c** Images of tadpoles 3-4 days after injection of embryos with LacZ (Control) or OLPVR1 mRNA before (Dark) and during illumination. **d** Light-induced responses of a single OLPVR1-expressing tadpole (n = 40 illuminations) were variable and consisted of a combination of tail flicking as in c, swimming or post-illumination body twitching (see Supplementary Note 3 & Supplementary Movies 1-5). OLPVR1-expressing tadpoles subjected to tubocurarine and MS-222 only showed tail flicking. **e** Delays of onset after light application, and offset after light switch-off, of behavioural response of OLPVR1 tadpoles. Numbers of observations are in parentheses (Error bars, SEM). Source data are provided as a Source Data file.

Calcium release is the trigger of muscle contraction. To test whether OLPVR1 can be expressed in living animals and produce light-dependent behavioural changes, we expressed OLPVR1 in amphibian *Xenopus laevis* tadpoles. Expression was achieved by injection of 1 ng OLPVR1 mRNA in one cell of two-cell stage *Xenopus* embryos. Testing was performed within 4 days of development after embryos had developed into free-swimming tadpoles. Results are illustrated in Fig. 5 (see also Supplementary Note 3 & Supplementary Movies 1-5). Illumination with 505-530 nm light produced distinct motion in half of OLPVR1-injected tadpoles while it had no effect in control tadpoles injected with ß-Gal mRNA. We did not quantify expression of OLPVR1 but we know from experiments with single oocytes that protein expression after mRNA microinjection is highly variable. In Fig. 5d we summarize in a Venn diagram the different behavioural responses observed in a series of 40 consecutive illuminations on a single tadpole. The responses to light were a combination of tail flicking, swimming and twitching. Tail flicking was the most frequent behaviour appearing in 83% of illumination instances. We further examined the onset and offset of the observed behaviours (Fig. 5e). Tail flicking started and ended within 2 s when light was switched on and off. Swimming started more slowly, within 6 s, and stopped after >15 s. The time frame of tail flicking is consistent with OLPVR1 light-induced Ca^2+^ release and muscle contraction. Since OLPVR1 expression was not targeted to any particular cell type, effects of OLPVR1-activation in different tissues other than muscle were not excluded in these experiments. To isolate events of muscle contraction we subjected a subset of OLPVR1-injected light-responsive tadpoles to the neuromuscular junction blocker tubocurarine, as well as MS-222/tricaine, an anesthetic blocking sensory-motor transmission. Four out of the five tadpoles tested retained light responsiveness (Fig. 5b) in the form of tail flicking, shown by the single behaviour observed upon 40 consecutive illuminations (Fig. 5e). These experiments suggest that tail flicking, but not swimming, is a direct result of triggering OLPVR1-mediated Ca^2+^ release in the muscle cells of the tadpoles.

## Discussion

Evidence in two different cell types demonstrates that native OLPVR1 localizes intracellularly in the ER and, upon light irradiation, mediates a rise in intracellular Ca^2+^ through release from IP_3_-dependent Ca^2+^ stores. Such so far unreported phenotype appears not to be an isolated outlier as it was shared by the two other related VCR1s tested, VirChR1 and TARA150 (Supplementary Fig. 9 & Supplementary Note 4).

How do VCR1s increase cytoplasmic Ca^2+^? Many rhodopsins are proton pumps and alkaline pH in the ER lumen inhibits Ca^2+^ uptake by the Ca^2+^-ATPase pump, thus promoting an increase in cytosolic Ca^2+^ ^28^_,_. OLPVR1 can also pump protons, but very weakly and in a direction that would instead acidify the ER lumen^8^. The most parsimonious mechanism would rather be for these proteins to serve as a Ca^2+^-release channel in the ER membrane. In oocytes and HEK293T cells, despite our many attempts, whole-cell currents could not be obtained to directly investigate ion selectivity. However, Ca^2+^ imaging of GCaMP6s-fused OLPVR1 strongly suggests direct Ca^2+^ permeation. The only recording of VCR1 currents comes from neuroblastoma cells^8^ where OLPVR1 did not express but VirChR1 expressed weakly, and sufficiently, at the surface membrane to show a Na^+^/K^+^ permeable channel blocked by extracellular Ca^2+^ above ~2 mM. This block by Ca^2+^ is significant because it ensures that, in the high-Ca^2+^ environments of algal hosts (seawater, hypersaline Organic Lake), any VR present at the outer surface membrane of algae would be inactive. For ER-localized VCR1s, this block would be negligible because the external face of the channel would face the ER lumen where Ca^2+^ concentration does not exceed hundreds of µM^29^. Furthermore, this apparent block at the plasma membrane might be a consequence of Ca^2+^ ions permeating at such low rates that they would not produce any measurable current. Such a mechanism would resemble the leaking of Ca^2+^ ions through voltage-dependent Na^+^ channels responsible for transient intracellular Ca^2+^ rise in neuronal axons^30^.

If animal rhodopsins natively reside in internal membranes^31^, it is accepted that microbial channelrhodopsins, such as *Chlamydomonas reinhardtii* ChR2^32^, natively localize and function at the surface membrane, characteristically in the plasma membrane fraction of specialized eyespot structures. Our results challenge this view by demonstrating that channelrhodopsins can have a biological function in internal membranes. When expressed in heterologous systems, microbial rhodopsins often accumulate in intracellular domains and have to be subjected to optimizations for surface membrane expression^33^. In *Xenopus* oocytes, we found that a sizeable fraction of ChR2 proteins is intracellular but illumination of ChR2-expressing oocytes did not elevate intracellular Ca^2+^, at least to levels sufficient to activate CaCCs. This suggests that internal ChR2, unlike VCR1s, is not associated with Ca^2+^ stores. In contrast, when ChR2 was engineered to target the ER, its photoactivation caused elevation of intracellular Ca^2+^ ^34^. Interestingly, in our conditions, ChR2-ER indeed localized in the ER but, contrary to OLPVR1, did not increase intracellular Ca^2+^ enough to trigger Ca^2+^-activated ion channels (Supplementary Fig. 10). Precise targeting of VCR1s appears to be a key factor in their ability to trigger Ca^2+^ release and fulfil their cellular function. Preferential localizations cannot be explained by already described retention or export signal sequences. The amino acid sequences of VCR1s when subjected to localization prediction tools^35^ showed no consistent result.

Organic Lake *phycodnavirus* is thought to infect prasinophytes or prymnesiophytes, members of unicellular algal flagellates, the most probable host being the prasinophyte *Pyramimonas*^36^. The viruses carrying VirChR1 and TARA150 are unidentified. Channelrhodopsins in better known algal organisms reside in the plasma membrane at the eyespot and are the precursor of a phototransduction cascade involving depolarization, voltage-dependent Ca^2+^ channels activation, Ca^2+^-induced Ca^2+^ release from intracellular stores, and eventually flagellar motion and phototaxis^9–11,37^. Such a scheme would be altered in infected algae, with VCR1s providing a direct link between irradiation and intracellular Ca^2+^, bypassing any electrical signalling. VCR1s could modify the behaviour of their hosts to favour virus replication, possibly conferring or enhancing phototaxis^4^. Hijacking intracellular Ca^2+^ dynamics is a strategy of many viruses. Notably, some Ca^2+^-permeable viroporins (virus-encoded ion channels) are targeted to the host ER membrane and modulate intracellular Ca^2+^ homeostasis to promote or prevent host apoptosis^38,39^. By analogy, VCR1s could also confer light-dependent protection against, or susceptibility to, apoptosis. Ultimately, VCR1s have the hallmarks to be main players in how giant viruses control phytoplankton populations and impact world nutrient cycles since they constitute an exogenous molecular transducer that responds to the host fundamental energy source – light – and connects it to the most ubiquitous cell signalling trigger – calcium.

The precise release of calcium from intracellular stores mediates a panoply of transduction cascades in cellular processes and pathologies such as gene expression, neurotransmitter release, hormone release, muscle contraction, host-pathogen interactions, tumor development or neurodegenerative disorders. Mobilization of intracellular calcium is already achievable by pharmacological approaches^21^. However, leveraging the spatiotemporal resolution of light delivery could enable more precise control of calcium release with potential advantages to our understanding of calcium signalling. The propensity of VCR1s to accumulate in internal storages with little or no sequence engineering and modulate calcium release, in a fine-tunable manner depending on the light intensity, makes them attractive optogenetic tools, with potential applications in the manipulation of numerous aspects of cell activity and, as demonstrated, of animal behaviour.

## Methods

### Ethical Statement

*Xenopus laevis* animal handling and experiments fully conformed with European regulations and were approved by the Ethics Committee of the *Commissariat à l’Energie Atomique et aux Energies Alternatives* (Ethics Approval #12-040). Authorization of the animal facility has been delivered by the regional administration (*Préfet de l’Isère, authorization # D 38 185 10 001*). Experiments on *Xenopus laevis* tadpoles were carried out in accordance with the European Community Guide for Care and Use of Laboratory Animals and approved by the “Comité d′éthique en expérimentation de Bordeaux”, N° 33011005-A.

### Molecular biology

For experiments in *Xenopus* oocytes and tadpoles the genes of OLPVR1 (GenBank: ADX06642), OLPVR2 (GenBank: ADX06595), TARA150 (GenBank: MAV65030 with additional N-terminal sequence MVGGSL), VirChR1 (TARA-146-SRF-0.22-3-C376786_1), and ChR2 (fused to mKate red fluorescent protein; kindly provided by Prof. Christoph Fahlke, Jülich, Germany) were subcloned in the pXOOM vector^40^, whilst the human muscarinic receptor M3 (GenBank: NP_001362914) and β-gal were subcloned in a pGEMHE vector^41^. Protein constructs OLPVR1_HB_ and ChR2_HB_ were fused at the extracellular N-terminal with the HiBiT tag (sequence: VSGWRLFKKIS) followed by a GSSGGS linker. For that purpose, the corresponding nucleotidic sequences were inserted by standard PCR after the start codon of the OLPVR1 and ChR2 genes. mRNA coding for all proteins was prepared in vitro using the mMessage mMACHINE T7 Transcription Kit (#AM1344, Invitrogen) and purified using the NucleoSpin RNA XS purification kit (#740902, Macherey-Nagel).

For experiments in HEK293T (#CRL11268, ATTC) the genes were subloned as follows: OLPVR1 in pIRES2-EGFP or pCMV, mouse TMEM16A (NP_848757), TMEM16B (NP_705817) in pmCherry-N1, and human SK1 (isoform 1, NP_001373903), OLPVR1-GFP, GCaMP6s^25^, OLPVR1-GCaMP6s and OLPVR1(K204Q)-GCaMP6s in pcDNA3.1. OLPVR1-GCaMP6s and OLPVR1(K204Q)-GCaMP6s were synthetized by GeneCust with a short linker (TAVATI) between the C-terminal of OLPVR1 and GCaMP6s.

Where noted, sequences were added at the N-terminus (HA, hemagglutinin epitope; SS, N-ter signal sequence of 25 amino acids from human nicotinic acetylcholine alpha 7 receptor subunit isoform 1^42^) and the C-terminus (MT, C-ter Golgi export trafficking signal sequence – KSRITSEGEYIPLDQIDINV – of Kir2.1 channels^43,44^).

### *Xenopus* oocytes

Ovary lobes were surgically removed from mature *Xenopus leavis* females, teased apart with forceps, and oocytes were isolated by enzymatic removal of follicular cells with collagenase^45,46^. Each oocyte was injected with 50 nl of water containing the indicated amount of mRNA in ng. Oocytes were injected with 2.5 ng of M3 mRNA, 7.5 ng of ChR2/ChR2_HB_ mRNA, and/or 7.5 to 30 ng of OLPVR1/OLPVR1_HB_, TARA150, VirChR1 mRNA (lower, as well as higher, amounts yielded smaller currents). Microinjected oocytes were incubated in the dark at 19 °C in modified Barth’s solution (in mM: 1 KCl, 0.82 MgSO_4_, 88 NaCl, 2.4 NaHCO_3_, 0.41 CaCl_2_, 0.3 Ca(NO_3_)_2_, 16 HEPES, pH 7.4) supplemented with 100 U/ml penicillin and streptomycin, 0.1 mg/ml gentamycin and 1 μM all-trans retinal (#R2500, Sigma-Aldrich). The oocytes were left to express proteins for 1 to 4 days before electrophysiological recordings were performed. Expression levels of ion channels in oocytes are notoriously variable, but less so when oocytes are from the same batch. Although we present averages from oocytes of different batches, we ascertained that our observations were valid when comparing oocytes from the same batches.

### XenoGlo: Surface expression measurements in oocytes

Briefly, this method is based on the formation of an active Nanoluciferase by the high-affinity complementation of its two parts, a short 11-aminoacid peptide (HiBiT) inserted as a tag in the target protein, and a larger soluble LgBiT protein.

Oocytes injected with mRNA coding for HiBiT-tagged proteins such as OLPVR1_HB_ and ChR2_HB_ were dispensed with the animal pole facing up in white round-bottom 96-well plates pre-filled with 100 μl of modified Barth’s solution in each well.

Surface luminescence measurements of oocytes were performed using the kit Nano-Glo HiBiT Extracellular Detection System (#N2421, Promega) by adding to each well 100 μl of the Nano-Glo HiBiT extracellular reagent. After 10 minutes of incubation, at 19 °C and 200 RPM, relative luminescence units were recorded for each well from a top optic with a focal height of 11 mm and a gain of 3500 using a CLARIOstar plate reader (BMG Labtech).

After surface luminescence measurements, oocytes were washed for ≈ 30 seconds in modified Barth’s solution with gentle agitation. Luminescence measurement of permeabilized oocytes was performed subsequently using the kit Nano-Glo HiBiT Lytic Detection System (#N3040, Promega) by applying an equivalent protocol as described above. Measurements after lysis yield estimates of the total expression of HiBiT-tagged proteins.

Surface and total expression of a given construct was measured in several batches of oocytes expressing that construct. For each batch, luminescence values of 3 oocytes before and after lysis were measured and averaged. The resulting values were used to calculate averages over several batches of surface and total expressions.

### Oocyte electrophysiology

Whole-cell currents were recorded with the two-electrode voltage clamp (TEVC) technique using a GeneClamp 500B Amplifier (Molecular Devices), a Digitizer Digidata 1440 A (Molecular Devices) and an eight-channel perfusion system with a manually operated controller from AutoMate Scientific. Some experiments where light stimulation was not needed were conducted with a HiClamp robot (MultiChannel System). Microelectrodes were filled with 3 M KCl and oocytes were perfused in the specified solutions. Bath solutions (composition in mM) were: ND96 (91 NaCl, 2 KCl, 1.8 CaCl_2_); ND96 0Ca (91 NaCl, 2 KCl); 94 K^+^ 100 Cl^-^ (94 KCl, 2 CaCl_2_); 94 Na^+^ 100 Cl^-^ (94 NaCl, 2 CaCl_2_); 94 K^+^ 10 Cl^-^ (4 KCl, 90 K Methanesulfonate, 2 CaCl_2_); 49 Ca^2+^ (49 CaCl_2_, 47 Glucose). All bath solutions had also 1 mM MgCl_2_ and 5 mM HEPES, and were adjusted to pH 7.4 with Tris or Citric acid, except for ND96 and ND96 0Ca where NaOH was used.

Currents were filtered at 3 kHz and sampled at 10 kHz.

Pulses of 505-nm light were applied with an OPTOLED LITE dual LED light source (Cairn Research Ltd; 5 A max LED current), coupled to a 1-mm optic fiber placed ~3 cm above the oocyte. In that setup, the light, applied with a single optical fiber, can reach at best half of the oocyte surface and can penetrate a few microns of the dense cytoplasm of the oocyte. We estimate that 90% of the light is absorbed within a layer of <40 µm below the surface. This rough estimate is based on data from Parker & Miledi^47^ who found a 1/10 attenuation of 350-nm UV light at a distance <10 µm below the oocyte surface, and from Ash et al^48^. who showed that 500-nm light penetrates 4-time deeper in tissues than 350-nm light. Assuming that illumination penetrates 40 µm below the surface, we calculate that ~5% of the intracellular volume of a 1.1-mm diameter oocyte is illuminated.

For oocytes injected with M3 mRNA, activation of the receptor was accomplished by perfusing 5 µM acetylcholine (#A6625, Sigma-Aldrich). In experiments with BAPTA_in_, each oocyte was injected with 50 nl of 40 mM-BAPTA (#2786, Tocris) solution and was left incubating for at least 30 min before recording. This yields an overall internal BAPTA concentration of ~3 mM in a stage VI oocyte of 1.1-mm diameter. In experiments where inhibitors were used the concentrations were as follows: 100 µM 2-APB (#1224, Tocris), 30 µM Ani9 (#6076, Tocris), 30 µM MONNA (#5770, Tocris) and 10 µM YM-254890 (#21910-1590, Tebu-BIO).

### *Xenopus* tadpoles

Three independent batches of *Xenopus laevis* embryos were obtained by inducing egg-laying of mature female frogs with 700 U of human chorionic gonadotropin (#CG10, Sigma-Aldrich) and bringing the eggs into contact with testis that had been teased open with forceps just before use. Fertilization was initiated by addition of distilled water to the egg mass. Eggs were dejellied 20 min after fertilization in a 2% cysteine-HCI pH 7.8 solution with gentle agitation for approximately 5 min and transferred into modified Barth’s solution^49^. Fertilized eggs were identified by the presence of a pigmented spot on the animal hemisphere of the egg at the sperm entry point. Embryos in stage two were identified by the fully cleaved embryo into two cells^50^. For each batch, at the two-cell stage, one cell of each embryo was injected with 250 pg lacZ (control) or 1 ng OLPVR1 mRNA.

### *Xenopus* tadpoles behaviour studies

Injected *Xenopus laevis* embryos were kept in the dark in modified Barth’s solution for 3 to 4 days until tadpoles reach stage 39 to 44. Individual tadpoles were placed in 35 mm dishes under a trinocular microscope mounted with an INFINITY8-2M Camera (Teledyne Lumera) for video recording (Infinity Analyzer v7.1.0). Tadpoles were subjected to 2-200 s green-light pulses using either OPTOLED LITE dual LED light source (Cairn Research Ltd; 5 Amax LED current, 505 nm) or Alonefire X004, (China, 510 ~ 530 nm). Where specified, tadpoles were incubated in the dark in modified Barth’s solution with 0.5 mM tubocurarine (#T2379, Sigma-Aldrich) and 0.02% MS-222 (#A5040, Sigma-Aldrich) for at least 20 min and checked for loss of muscle tone by flowing media into the dish with a pipette. The concentration of each compound was empirically and individually optimized on non-injected tadpoles. Once immobilized the tadpoles were subjected once again to light pulses as previously described. After the experiments, tadpoles were retransferred to modified Barth’s solution and checked for recovery of muscle tone to confirm their survival.

### Mammalian cells electrophysiology

HEK293T cells were maintained in DMEM supplemented with 10% FBS in 35-mm dishes. At 70–80% confluency, cells were transiently transfected using the calcium phosphate method with 1.5 µg of OLPVR1 DNA and 2.1 µg of TMEM16A, TMEM16B or SK1 DNA, and seeded on 35-mm diameter plates.

Whole-cell patch-clamp recordings in HEK293T cells were performed 1-2 days after transfection. Recordings were performed in voltage-clamp mode using an Axopatch 200B (Molecular Devices) amplifier. The standard bath solution contained (in mM): 150 NaCl, 5 KCl, 10 HEPES (pH 7.4) and 2 CaCl_2_. The standard pipette solution contained (in mM): 155 KCl, 3 MgCl_2_, 10 HEPES (pH 7.3). Unless otherwise specified, it also contained 10 µM EGTA. EGTA in the pipette was used to chelate contaminant Ca^2+^. From specification sheets of salt (Sigma-Aldrich) we estimated contaminant Ca^2+^ to be ~3 µM, and a predicted (Alex software^51^) free Ca^2+^ of 114 nM in 10 µM EGTA. Pulses of 505 nm-light were applied using a Thorlabs M505L4 LED mounted on a Zeiss Axiovert 200 M microscope under a Zeiss LD Achroplan 40X/0.6 Korr 440864 objective. Signals were filtered at 10 kHz and sampled at 20 kHz.

### Mammalian cells confocal fluorescence imaging

HEK293T cells were seeded in 25-mm poly-L-lysine-treated coverslips and transfected at 70–80% confluency using Lipofectamine 2000 (ThermoFischer Scientific) with DNA for OLPVR1 fused to GFP at the C-terminal in pcDNA3.1, and pDsRed2-ER vector (Clontech, #632409) in a 6:1 ratio. After one day of expression, the cells were incubated for 30 min at 37 °C in standard bath solution with Hoescht 33342 (1/5000 dilution) and CellMask Deep Red (1/1000 dilution; #C10046 ThermoFischer Scientific). Hoescht 33342 was applied to stain the cell nucleus and CellMask Deep Red to stain the plasma membrane. pDsRed2-ER expression was used to label the ER. After the incubation, the cells were washed twice with standard bath solution and imaged immediately. Images were acquired using Zeiss Zen Black 2.3 software through the objective Plan-Apochromat 63x/1.40 Oil DIC M27 of a Zeiss LSM 710 Laser Scanning Confocal Microscope equipped with the lasers DPSS 561-10 nm, Argon 488 nm, Diode 405-30 nm and Helium Neon 633 nm, as well as two descanned GaAsp detectors ([570-610 nm], [500-550 nm]) and a descanned PMT detector set to 415-485 nm or 642-740 nm. Composite images result from channel merging with ImageJ software.

### Mammalian cells calcium imaging

HEK293T were obtained and authenticated by ATCC #CRL-11268, used regularly in recent years and not further authenticated. Cells were tested regularly and confirmed negative for mycoplasma contamination using the MycoAlert kit (Lonza). Cells were seeded in 35-mm culture plates and transfected at 70–80% confluency using jetOPTIMUS reagent (Polyplus) 1:1 with a total of 1 µg of DNA. After one day of expression, cells were bathed in standard bath solution (described above) and videoimaging was performed using Micro-Manager 1.4.21 software in a Zeiss Axiovert 200 M epifluorescence microscope mounted with a Thorlabs M470L4 Led, a Zeiss Filter set 44 (BP 475/40, FT500, BP 530/50), a Zeiss LD Achroplan 40X/0.6 Korr 440864 objective and an Andor Zyla sCMOS camera. Acquisition was obtained at 10 fps. Fluorescent values were extracted from individual cells using ImageJ 1.54d software. Confocal images were obtained from cells seeded in 25-mm poly-L-lysine-treated coverslips, using MetaMorph 7.10.5.476 software, in a Nikon TiE microscope under a 40X objective with Ilas2 illumination module set to 488 nm (4.5%), a Yokogawa CSU W1 confocal scanner unit and a Andor iXon Life 888 (emCCD) camera. When specified, cells were incubated for 2 h with 15 µM BAPTA-AM (#2787,Tocris) in DMEM supplemented with 10% FBS at 37 °C and then bathed in standard bath solution with the same concentration of BAPTA-AM during imaging.

### Data analysis

For TEVC and patch-clamp recordings, data acquisition was performed using pClamp software (Molecular Devices). HEK cells data were analyzed with pClamp. Oocytes data were transferred to Microsoft Excel after undersampling to 10 or 100 Hz. Annotation, plotting, and analysis of the current recordings were automated by the use of Excel add-ins, including eeTEVC^46^. Slow fluctuations of the baseline of the current signal were removed by interactive fitting of the baseline with a spline curve and subtraction of this fit from the signal. For luminescence experiments, unless otherwise noted, data points were blank-corrected and values shown represent the average of triplicates. For fluorescence measurements, traces were background subtracted first before any other computation. For determination of half times of fluorescence changes, each trace was fitted by nonlinear regression with an unconstrained exponential curve.

### Statistics & Reproducibility

No statistical method was used to predetermine sample size. For each independent experiment sample size was maximized based on experimental logistic constraints. No technical replicates were used. For each experiment, repetitions were performed until the inclusion of the last data batch would not change the variance of the total sample significantly. Experiments were repeated independently at least twice except for: the experiment in Fig. 5b which was performed on 5 independent tadpoles from 1 single batch; the experiments in Figs. 1a, 1g (BAPTA_in_), Sup Fig. 1c (BAPTA_in_), Sup Fig. 1 g,h, Sup Fig 6 (where n < 7) and Sup Fig 9 b (ChR2, TARA150) that were performed in one single batch of oocytes; the experiments in Fig. 3b and Sup Fig. 5 that were performed in cells from one single transfection. The investigators were not blinded to allocation during experiments and outcome assessment since all experiments were performed by isolated researchers without additional staff.

Statistical significance analysis was done using GraphPad Prism 8.0.1. Tests and values are indicated in the figure legends alongside the data. For maximal fluorescence changes and half time of fluorescence change determination, outliers were identified and removed using the ROUT (robust regression and outlier removal) method with a coefficient Q of 1%.

### Reporting summary

Further information on research design is available in the Nature Portfolio Reporting Summary linked to this article.

### Supplementary information

Supplementary Info

Peer Review File

Description of Additional Supplementary Files

Supplementary Movie 1

Supplementary Movie 2

Supplementary Movie 3

Supplementary Movie 4

Supplementary Movie 5

Reporting Summary

### Source data

Source Data

## Data Availability

Source data are provided as a Source Data file. Source data are provided with this paper.
