# Supplementary material for: Hijacking of internal calcium dynamics by intracellularly residing viral rhodopsins
Source: Nat Commun. 2024 Jan 2;15:65. doi: 10.1038/s41467-023-44548-6 (PMC10761956; doi:10.1038/s41467-023-44548-6)
Supplement: Supplementary file 1 — Supplementary Info [file 41467_2023_44548_MOESM1_ESM.pdf]

Supplementary Information

Hijacking of internal calcium dynamics by intracellularly residing viral rhodopsins

Ana-Sofia Eria-Oliveira[1,2,3,4], Mathilde Folacci[1,3,#], Anne Amandine Chassot[2,3,4], Sandrine Fedou[5], Nadine Thézé[5], Dmitrii Zabelskii[6], Alexey Alekseev[7,8], Ernst Bamberg[9], Valentin Gordeliy[1,10], Guillaume Sandoz[2,3,4,*], Michel Vivaudou[1,3,*]

[1] Univ. Grenoble Alpes, CEA, CNRS, IBS; Grenoble, France.

[2] Université Côte d’Azur, CNRS, INSERM, iBV; Nice, France.

[3] Laboratories of Excellence, Ion Channel Science and Therapeutics; Nice, France.

[4] Fédération Hospitalo-Universitaire InovPain, Cote d'Azur University, University Hospital Center Nice; Nice, France.

[5] Univ. Bordeaux, Inserm, BRIC, UMR 1312; Bordeaux, France.

[6] European XFEL; Schenefeld, Germany.

[7] Advanced Optogenes Group, Institute for Auditory Neuroscience and InnerEarLab, University Medical Center Göttingen; Göttingen, Germany.

[8] Cluster of Excellence "Multiscale Bioimaging: from Molecular Machines to Networks of Excitable Cells" (MBExC), University of Göttingen; Göttingen, Germany.

[9] Max Planck Institute of Biophysics; Frankfurt am Main, Germany.

[10] Institute of Biological Information Processing (IBI-7: Structural Biochemistry), Forschungszentrum Jülich; Jülich, Germany.

[#] Present address: Department of Biomedicine, Aarhus University, Aarhus, Denmark.

* Corresponding authors. Email: vivaudou.lab@gmail.com, sandoz@unice.fr

Supplementary Notes

Supplementary Note 1: Multiple sequence alignment of VCR1s

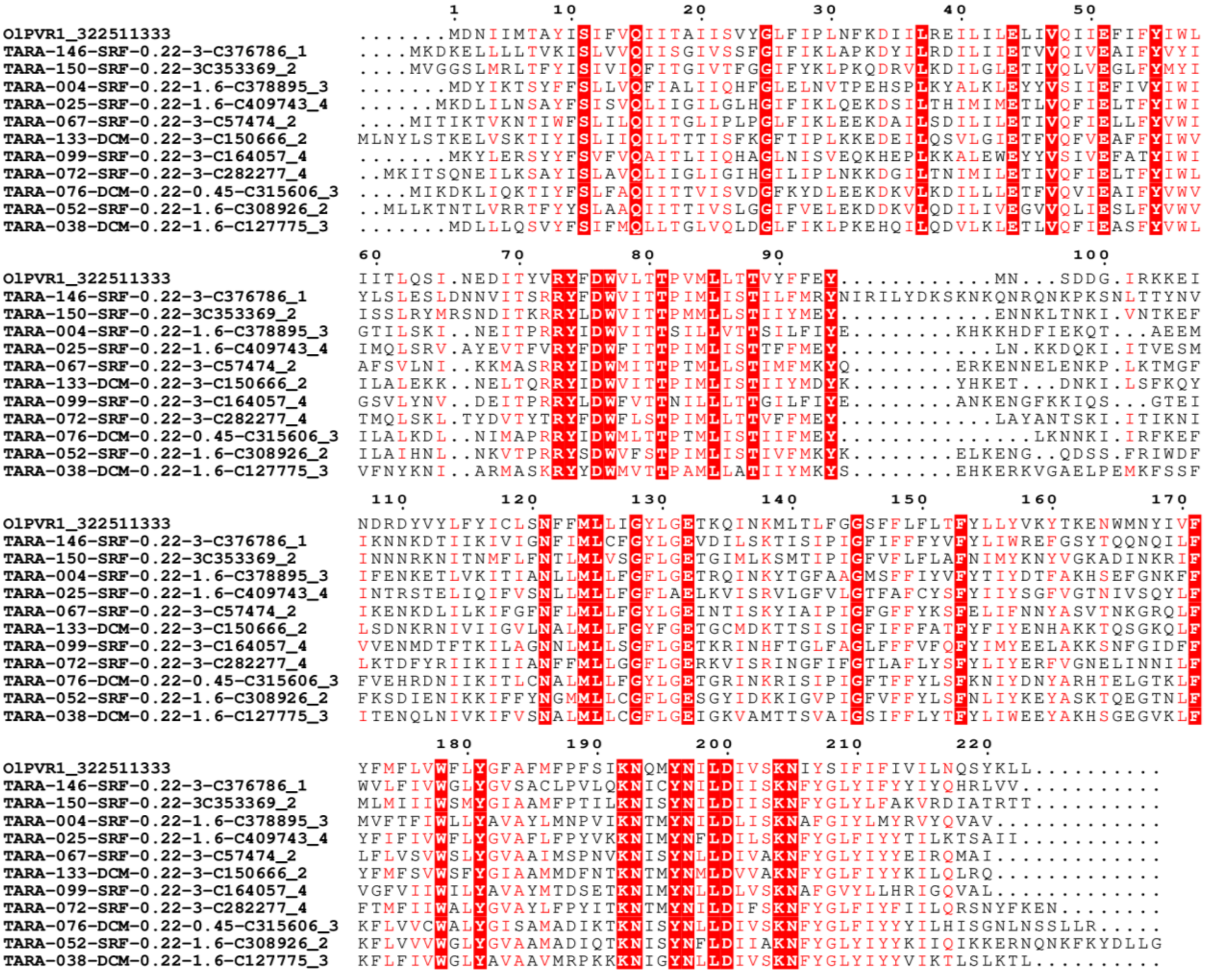

OLPVR1 sequence (OLPVR1_322511333) was used as the reference sequence. VirChR1 (TARA-146-SRF-0.22-3-C376786_1) and TARA-150 (TARA-150-SRF-0.22-3C353369_2) sequences are shown. Highly (>70%) and fully conserved (100%) residues are shown in red font and red highlight, respectively.

Supplementary Note 2: Promoting surface expression of OLPVR1

Although exogenous membrane proteins have usually better surface expression in *Xenopus* oocytes than other systems[1], engineering surface-addressed OLPVR1 proved difficult (Supplementary Fig. 6). Only one construct, the DOR=OLPVR1 fusion, displayed significant, though modest, surface expression, but its analysis was not pursued because (1) its photocurrents were too small (<5 nA) and (2) it functionally diverged from OLPVR1 as it did not induce Ca^{2+} release despite being highly enriched intracellularly.

The Organic Lake phycodnavirus genome where the OLPVR1 gene was found features another rhodopsin gene coding for OLPVR2[2]. In oocytes, OLPVR2 did not express at the surface and expressed only weakly (14-fold less than OLPVR1) intracellularly (Supplementary Fig. 6). We were unable to record any electrophysiological activity associated with OLPVR2, native or with added signal sequences, in oocytes. Nor did we find that coexpression of OLPVR2 affected the properties of the photocurrents elicited by OLPVR1.

Supplementary Note 3: Information on the illumination protocol used in the supplementary movies

Both green and red illuminations on the supplementary movies were performed under continuous light application using an Alonefire X004 lamp that emits both continuous green (510-530 nm) or red light (600-720 nm). In the red-light illumination Supplementary Movie 2, continuous red light appears as flashes. However, that is only an optical illusion, a consequence of the longer wavelength of red light in combination with the camera and video acquisition sampling settings, akin to the 'wagon-wheel' stroboscopic effect.

Supplementary Note 4: Other viral rhodopsins

OLPVR1 belongs to viral rhodopsin group 1. We tested two proteins from the same group, identified in Tara Ocean Foundation marine genomic data by database mining and of unknown marine origin[3,4] : VirChR1 and TARA150. As shown in Supplementary Fig. 9, TARA150 and VirChR1 generated photocurrents that resembled the CaCCs currents generated by OLPVR1. The tested constructs were modified by addition of SS and/or MT sequences at their termini (See Methods) as these gave more robust responses. Such modifications, although supposed to promote surface expression, did not change the expression profile when used with OLPVR1 (Supplementary Fig. 9). We further tested VirChR1 in HEK293T and observed light responses akin to those seen with OPLVR1, i.e., absence of photocurrents in VirChR1-transfected cells and large photocurrents in cells co-transfected with TMEM16A (Supplementary Fig. 9c&d). In these experiments we used the same modified protein (HA-VirChR1-MT) as in a previous study using SH-SY5Y human neuroblastoma cells[3]. In those cells, VirChR1, but not OLPVR1, produced detectable whole-cell currents indicative of a Na^+/K^+ permeable channel downregulated by external Ca^{2+} higher than ~1 mM. Because the pipette contained 10 mM EGTA, any long-range process involving intracellular calcium was suppressed.

Supplementary Figures

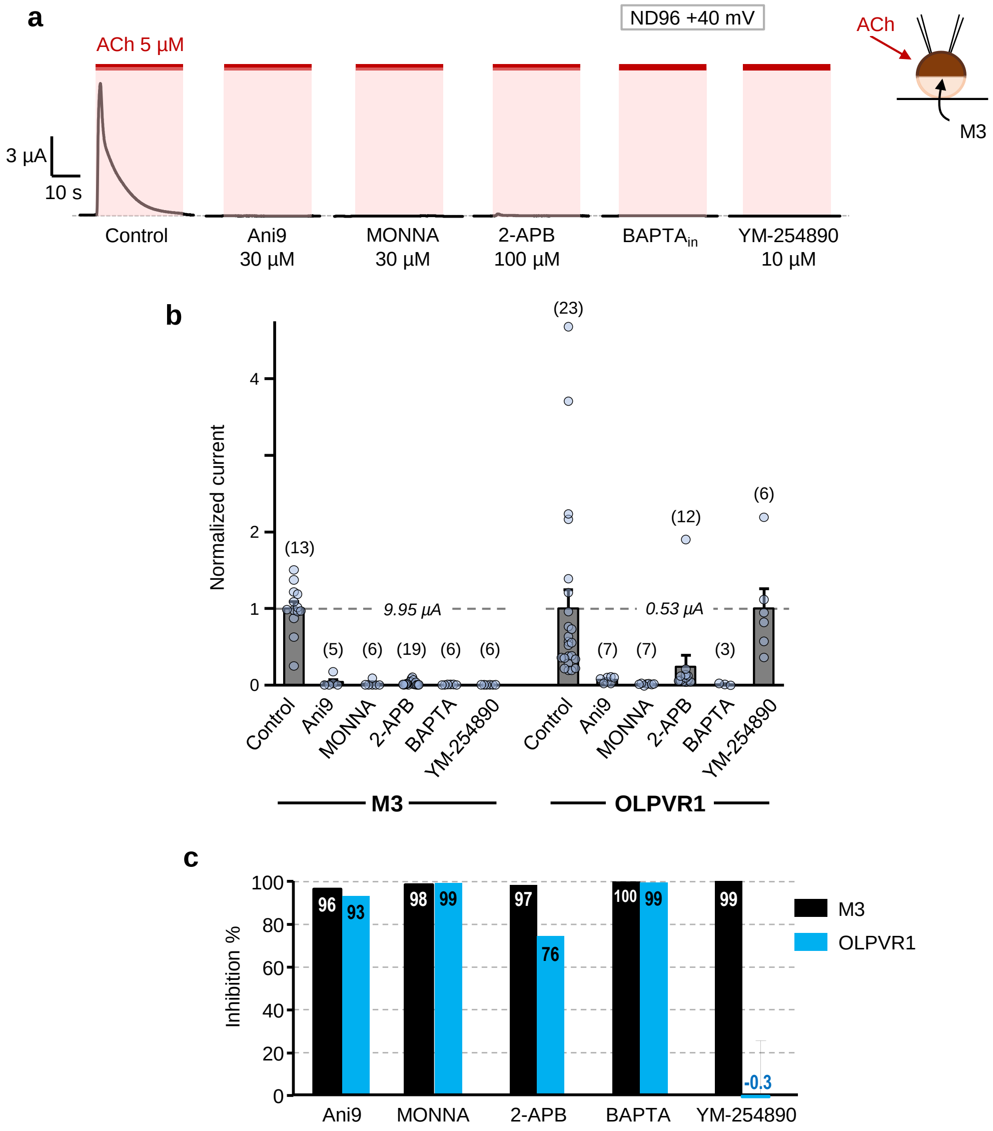

Supplementary Figure 1. Comparison of the effects of inhibitors on OLPVR1 photocurrents and M3-mediated currents.

a Representative currents elicited by 5 µM ACh in oocytes injected with 2.5 ng RNA coding for the Gq-coupled muscarinic M3 receptor. Bath solution = ND96, voltage = +40 mV. Oocytes were incubated in ND96 solution containing no inhibitor (Control), 30 µM Ani9, 30 µM MONNA, 100 µM 2-APB, or 10 µM YM-254890. Duration of incubation was 10’ for YM-254890, 60’ otherwise. BAPTA designates oocytes injected with 50 nl of a 40-mM BAPTA solution and left 60’ in ND96 solution prior to recording. The expected intracellular BAPTA concentration of BAPTA-injected oocytes is 3-4 mM for oocytes of diameter 1-1.1 mm.

b Normalized average currents measured in absence (Control) and in presence of the specified inhibitors in the same conditions as panel a for M3 and OLPVR1 injected oocytes. For M3, all compounds caused a significant inhibition (Student t-test; $p<1x10^{-7}$). For OLPVR1, Ani9, MONNA, and BAPTA caused a significant inhibition ($p<5x10^{-4}$), YM-254890 had no effect (p=0.64), and 2-APB had intermediate effect (p=0.012). Numbers of oocytes tested are in parentheses. The value of 1 corresponds to 9.95 µA for M3, and 0.53 µA for OLPVR1.

c Estimates of inhibition of M3-induced currents in the conditions of panel a (M3; Black bars) and of OLPVR1 photocurrents measured at +40 mV in ND96 solution (OLPVR1; Blue bars) calculated from the data in panel b. Values, specified in or above the bars, are calculated using solely the mean currents in Control and those in the presence of the indicated compounds. Tests in other solutions and/or at different voltages gave similar results. Source data are provided as a Source Data file.

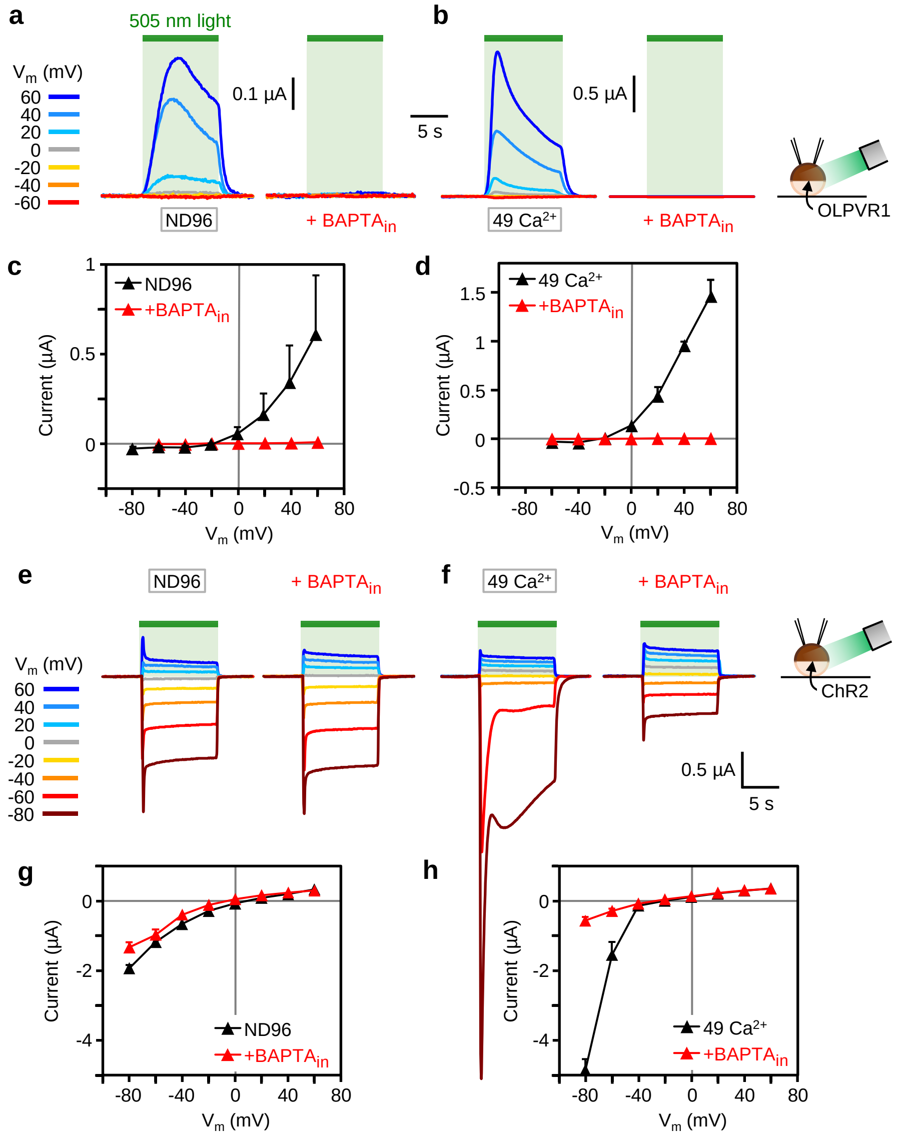

Supplementary Figure 2. Unlike ChR2, OLPVR1 photocurrents are abrogated by intracellular injection of BAPTA.

a Representative photocurrents from OLPVR1-expressing oocytes (7.5 ng RNA) with and without intracellular injection of BAPTA ($BAPTA_{in}$) in ND96 bath solution (which includes 1.8 mM Ca^{2+}).

b Same in 49 Ca^{2+} bath solution.

c Average peak photocurrent vs voltage in ND96 solution with (n=3) and without (n=9) injected BAPTA.

d Same in 49 Ca^{2+} bath solution with (n=4) and without (n=5) injected BAPTA.

e, f Same as panels a and b, but with an oocyte expressing ChR2 (7.5 ng RNA). All traces are from the same oocyte.

g, h Same as panels c and d, but with ChR2 in ND96 solution with (n=5) and without (n=4) injected BAPTA, and in 49 Ca^{2+} bath solution with (n=5) and without (n=4) injected BAPTA. (Error bars, SEM). Source data are provided as a Source Data file.

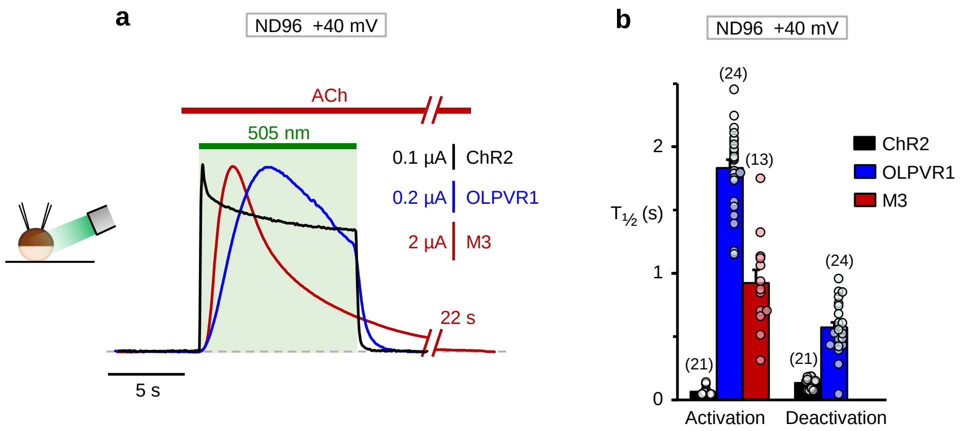

Supplementary Figure 3. OLPVR1 photocurrents activates slowly upon illumination.

a Representative currents elicited by illumination of oocytes expressing OLPVR1 (blue trace) or ChR2 (black trace), or by application of 5 µM ACh to an oocyte expressing M3 receptors (brown trace; break in the trace represents 22 s of omitted data). Superimposed traces were scaled vertically to the same size, vertical scale bars for each trace are indicated. Bath solution = ND96 solution, voltage = +40 mV.

b Average half-times of current activation upon light or ACh application, and deactivation upon light switch-off. Conditions were as in panel a; one data point/oocyte. The values for M3 were calculated as the time interval between the onset of the increase in current after ACh application and the time where current reached 50% of its peak value. They therefore do not include the latency inherent to bath application of agonists to oocytes. The time resolution of the recordings is ~50 ms so that the measured activation and deactivation $T_{½}$ of ChR2 of 65±6 and 135±9 ms, respectively, are upper estimates of the actual values. Numbers of oocytes are in parentheses (Error bars, SEM).

Source data are provided as a Source Data file.

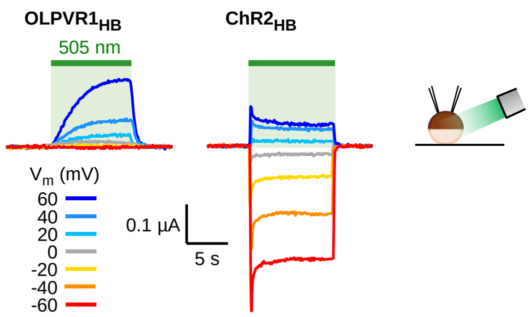

Supplementary Figure 4. HiBit tag does not modify the function of rhodopsins.

Representative photocurrents of HiBit-tagged OLPVR1 ($OLPVR1_{HB}$; 30 ng RNA/oocyte) and ChR2 ($ChR2_{HB}$; 7.5 ng RNA/oocyte) in ND96 solution. Such currents were recorded in 7 (OLPVR1) and 3 (ChR2) oocytes.

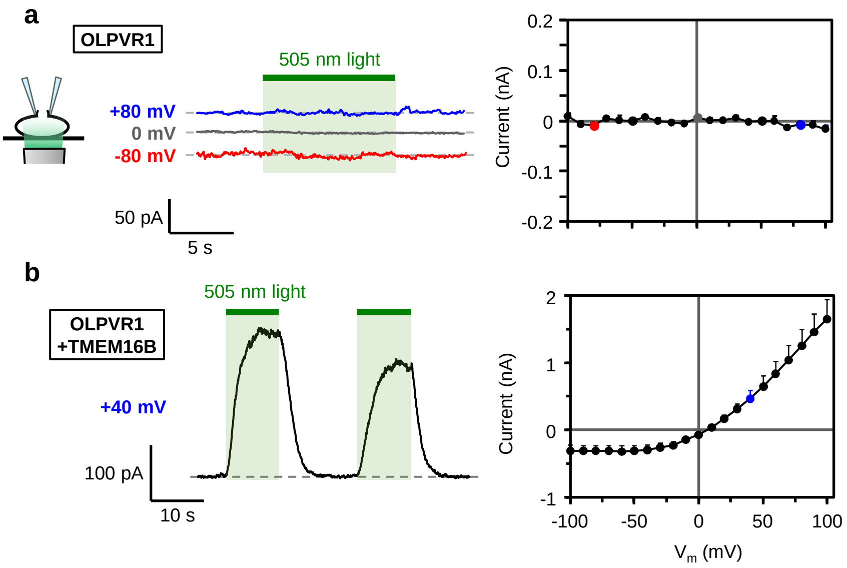

Supplementary Figure 5. OLPVR1 activates surface Ca^{2+}-activated TMEM16B channels in mammalian cells through release of intracellular calcium.

Data were obtained from HEK293T cells transfected with OLPVR1 alone (**a**) or with OLPVR1 and TMEM16B (**b**).

Left panels : Representative current responses to illumination (green bars, 505 nm, 10 s). Cells were held at the indicated voltages.

Right panels: Light-induced current (peak current elicited by first illumination – current before illumination) vs. voltage. Currents were elicited by voltage ramps from -100 to +100 mV (400 ms duration) repeated every second. n=7 for panel a, n=6 for panel b (Error bars, SEM). The bath solution contained (in mM): 150 NaCl, 5 KCl, 2 $CaCl_2$, and 10 HEPES (pH 7.4). The pipette solution contained (in mM): 155 KCl, 3 $MgCl_2$, 10 HEPES (pH 7.3) and 10 µM EGTA.

Source data are provided as a Source Data file.

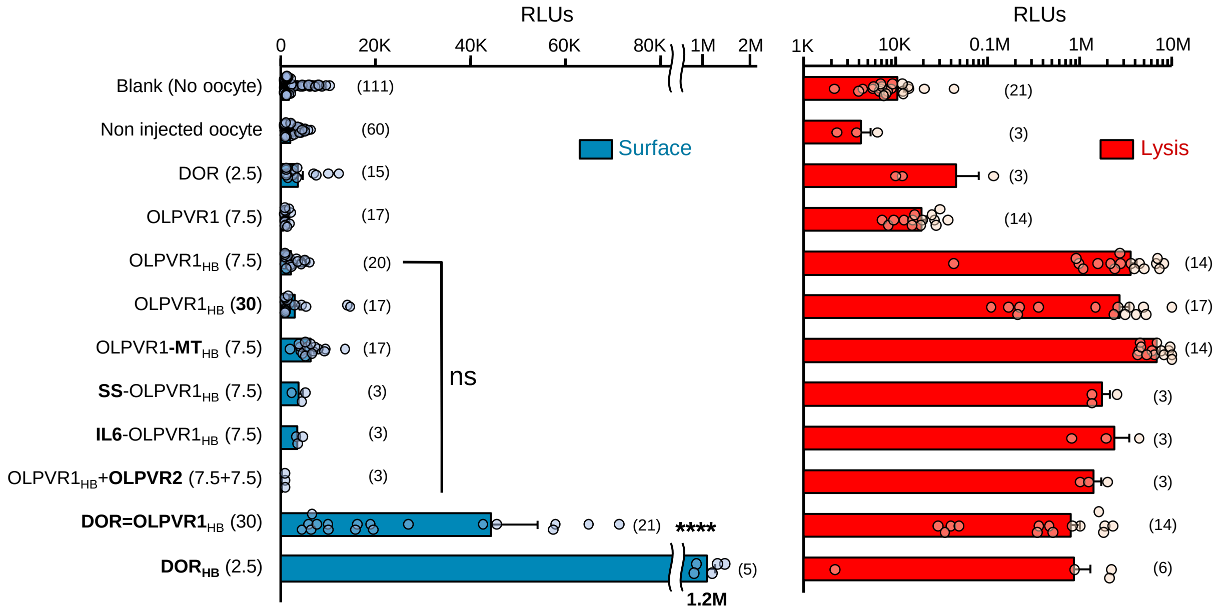

Supplementary Figure 6. Surface expression of various OLPVR1 constructs.

Mean luminescence (left: surface values, right: values after lysis) recorded in control conditions (no oocyte and non-injected oocytes) or from RNA-injected oocytes. Luminescence values were not blank corrected and averages were calculated from all oocytes tested. Amounts of RNA coding for specified proteins, in ng per oocyte, are indicated in parentheses. HB suffix indicated the presence of a HiBiT tag at the N-terminus. Oocytes were maintained in ND96 solution supplemented with 1 µM all-trans-retinal and tested 24-72h after injection. Increasing the amount of OLPVR1 RNA injected per oocyte from 7.5 to 30 ng did not affect surface expression. Modifying the protein by inserting signal sequences did not improve surface expression (MT, Golgi export trafficking signal of Kir2.1 potassium channel; SS, Signal sequence of human nicotinic acetylcholine alpha 7 receptor subunit; IL6, Interleukin 6 secretion signal sequence). Coexpressing OLPVR1 with viral rhodopsin OLPVR2 did not improve surface expression. However, the fusion DOR=OLPVR1 (30 ng) had a surface expression 15-fold higher than OLPVR1 (30 ng), but still 25-fold lower than DOR alone (DOR, δ-opioid receptor). **** $P<0.0001$; ns (not significant), $P>0.9999$; one-way ANOVA followed by Dunnett's multiple comparison test against the control OLPVR1 (7.5 ng). Numbers of oocytes are in parentheses (Error bars, SEM).

Source data are provided as a Source Data file.

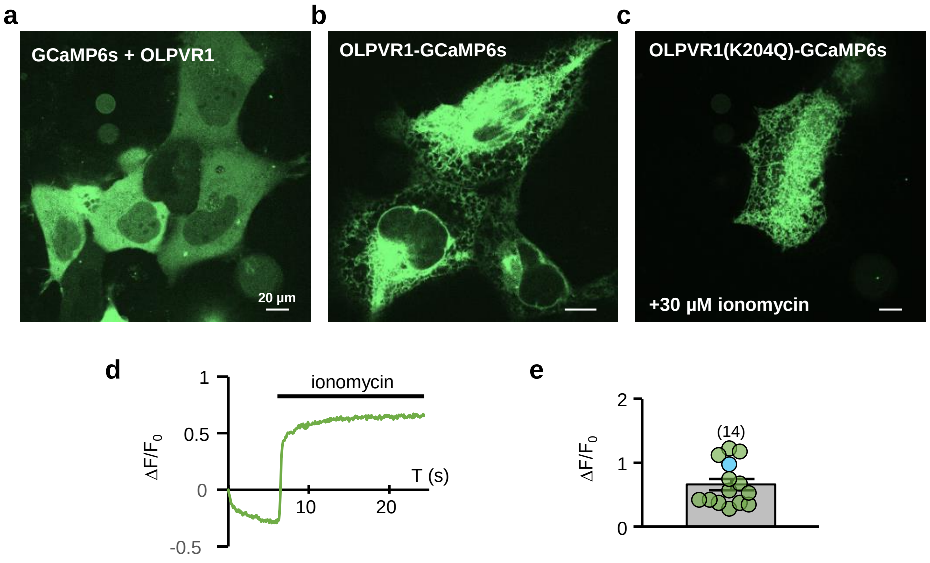

Supplementary Figure 7. GCaMP6s and OLPVR1-GCaMP6s have distinct cell expression patterns. **a, b** Confocal images of HEK293T cells transfected with plasmids for expression of (**a**) GCaMP6s and wtOLPVR1, (**b**) OLPVR1-GCaMP6s, and (**c**) OLPVR1(K204Q)-GCaMP6s incubated with 30 μM ionomycin.

d Time course of fluorescence change from a OLPVR1(K204Q)-GCaMP6s–transfected cell upon application of 30 μM ionomycin. Images are representative of 12 cells from 2 independent transfections.

e Average change in fluorescence of OLPVR1(K204Q)-GCaMP6s–transfected cells after application of 30 μM ionomycin. Results are pooled from 2 independent transfections. Number of experiments is in parentheses (Error bars, SEM). Blue point indicates the value from trace in d. GCamP6s has a cytoplasmic distribution while OLPVR1-GCaMP6s expresses at the ER. The K204Q mutation eliminates the formation of the Schiff base between lysine K204 and retinal, impeding the function of the rhodopsin. The mutation did not change the expression pattern of OLPVR1-GCaMP6s or the function of the GCaMP6s sensor, which still displayed fluorescence upon the increase of intracellular Ca^{2+} elicited by ionomycin.

Source data are provided as a Source Data file.

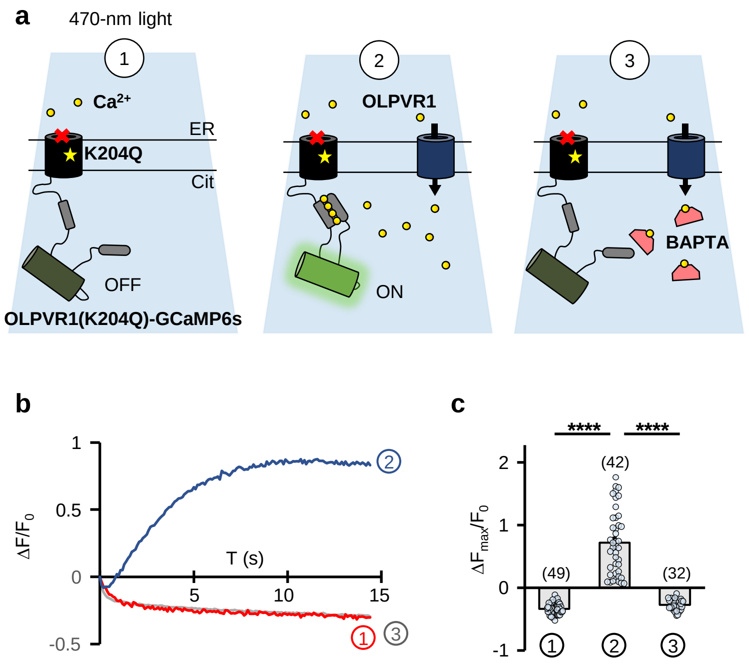

Supplementary Figure 8. BAPTA-AM chelates Ca^{2+} released from neighboring proteins.

a Schematic representation of the calcium-imaging conditions tested. HEK293T cells were transfected with OLPVR1(K204Q)-GCaMP6s, alone (1) or together with wild-type OLPVR1, and imaged in an epifluorescence microscope under 470-nm light in the absence (2) or presence (3) of 15 µM BAPTA-AM.

b Time courses of fluorescence changes upon light application of cells corresponding to the conditions in a.

c Average maximal fluorescence changes upon light application of cells from at least 3 independent transfections corresponding to the conditions in a. Number of experiments is in parentheses (Error bars, SEM). One-way ANOVA, Dunnett's multiple comparisons test **** $p<0.0001$, F (DFn 2, DFd 120) = 153. The K204Q mutation eliminates OLPVR1 light sensitivity without altering its expression (Fig. S7). Incubation of the cells with 15 µM BAPTA-AM for 2h eliminated the fluorescence signal due to Ca^{2+} influx from nearby OLPVR1 proteins, demonstrating that the conditions tested allow to identify the site of Ca^{2+} entry from the ER, i.e., whether Ca^{2+} is released by nearby proteins (chelated by BAPTA) or directly through the protein attached to the calcium sensor (not chelated by BAPTA).

Source data are provided as a Source Data file.

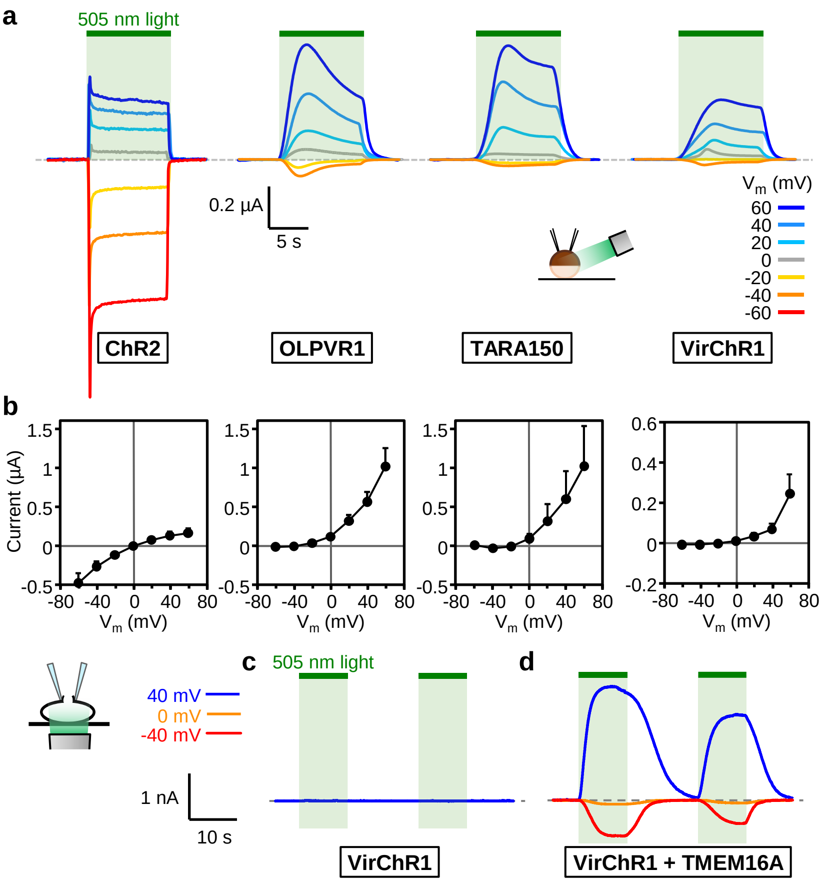

Supplementary Figure 9. Photo-induced currents of members of the VR1 family have a similar profile which is distinct from that of ChR2.

a Representative recordings in 94 K^+ 100 Cl^- solution from oocytes expressing, from left to right, ChR2 (7.5 ng RNA), and the viral rhodopsins OLPVR1, TARA150 (construct SS-TARA150), and VirChR1 (construct SS-VirChR1-MT) (30 ng).

b Current-voltage relationships of currents measured after 10 seconds of illumination, in KCl solution. (n=10 for OLPVR1, n=3 otherwise; Error bars, SEM).

c Representative whole-cell patch clamp recordings from a HEK293T transfected with HA-VirChR1-MT. Standard bath and pipette solutions.

d *Idem* from a cell co-transfected with VirChR1 (construct HA-VirChR1-MT) and TMEM16A.

Source data are provided as a Source Data file.

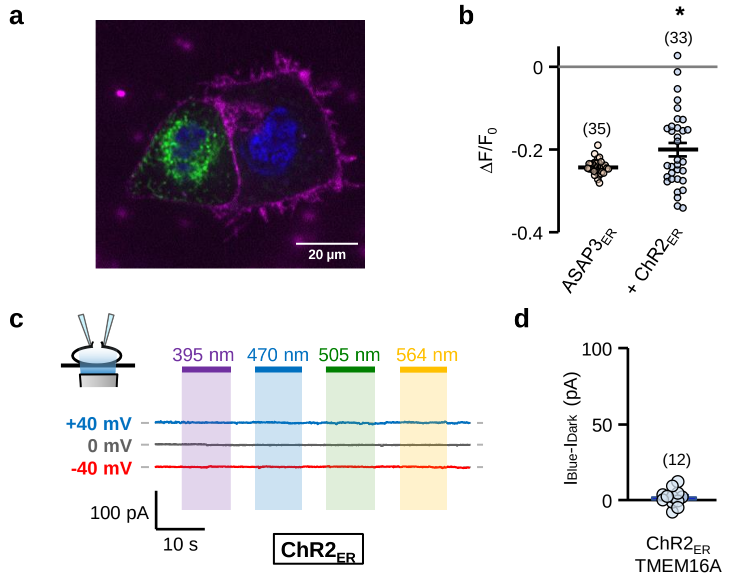

Supplementary Figure 10. ChR2ER localizes at the ER where it induces a change in voltage upon illumination, but does not increase intracellular Ca^{2+} enough to activate TMEM16A channels.

a Confocal images of HEK293T cells transfected with plasmids for expression of ChR2ER fused to a fluorescent protein (green). The nucleus is in blue and the plasma membrane in magenta. Image is representative of 12 cells from two independent transfections.

b Average maximal fluorescence changes of cells transfected with fluorescent voltage sensor ASAP3ER alone or with ChR2ER upon 470 nm-light application. Numbers of experiments are in parentheses (Error bars, SEM). Results are pooled from 3 independent transfections. Two-tailed t-test with Welch's correction (t=2.7, df=33.5, p=0.011), Variance *F* test (*F*=41.9, DFn=32, Dfd=33, p=<0.0001).

c Representative whole-cell patch clamp recordings from a HEK293T co-transfected with ChR2ER and TMEM16A under illumination with different wavelengths. Standard bath and pipette solutions (10 µM EGTA). Dashed lines represent the baseline at 0 current.

d Average current induced by 470-nm light measured at +40 mV as in panel c. Results are pooled from 2 independent transfections. Number of experiments is in parentheses (Error bars, SEM). Source data are provided as a Source Data file.

Supplementary References

1. Yang, K., Fang, K., Fromondi, L. & Chan, K. W. Low temperature completely rescues the function of two misfolded KATP channel disease-mutants. *FEBS Letters* **579**, 4113–4118 (2005).
2. Bratanov, D. *et al.* Unique structure and function of viral rhodopsins. *Nat Commun* **10**, 4939 (2019).
3. Zabelskii, D. *et al.* Viral rhodopsins 1 are an unique family of light-gated cation channels. *Nat Commun* **11**, 5707 (2020).
4. Karsenti, E. *et al.* A holistic approach to marine eco-systems biology. *PLoS Biol* **9**, e1001177 (2011).
