# Supplementary material for: Hijacking of internal calcium dynamics by intracellularly residing viral rhodopsins
Source: Nat Commun. 2024 Jan 2;15:65. doi: 10.1038/s41467-023-44548-6 (PMC10761956; doi:10.1038/s41467-023-44548-6)
Supplement: Supplementary file 3 — Description of Additional Supplementary Files [file 41467_2023_44548_MOESM3_ESM.pdf]

<u>**Description of Additional Supplementary Files**</u>

Supplementary Movie 1
OLPVR1-injected tadpole swims in response to green light
File: SV1.mp4
Tadpole 4 days after injection of 1 ng OLPVR1 RNA.
Test: 1 application (~30 s) of green light (510-530 nm) Result = swimming.

Supplementary Movie 2
OLPVR1-injected tadpole does not respond to red light
File: SV2.mp4
Tadpole 4 days after injection of 1 ng OLPVR1 RNA.
Test: 4 applications (~5 s) of red light (600-720 nm). Result = no response.

Supplementary Movie 3
OLPVR1-injected tadpole flicks tail and twitches in response to green light
File: SV3.mp4
Tadpole 4 days after injection of 1 ng OLPVR1 RNA.
Test: 4 applications (~2 s) of green light (510-530 nm). Result = tail flicking/twitching

Supplementary Movie 4
OLPVR1-injected tadpole in the presence of tubocurarine and tricaine (MS-222) flicks tail in response to green light
File: SV4.mp4
Tadpole 4 days after injection of 1 ng OLPVR1 RNA.
Test:1 application (~8 s) of green light (510-530 nm) in 0.5 mM tubocurarine and 0.02% tricaine. Result = tail flicking.

Supplementary Movie 5
LacZ-injected tadpole does not respond to green light
File: SV5.mp4
Tadpole 4 days after injection of 1 ng LacZ RNA.
Test: 1 prolonged application (~35 s) of green light (510-530 nm). Result = no response.
