# Supplementary material for: Hijacking of internal calcium dynamics by intracellularly residing viral rhodopsins
Source: Nat Commun. 2024 Jan 2;15:65. doi: 10.1038/s41467-023-44548-6 (PMC10761956; doi:10.1038/s41467-023-44548-6)
Supplement: Supplementary file 9 — Reporting Summary [file 41467_2023_44548_MOESM9_ESM.pdf]

nature portfolio

Corresponding author(s): Michel Vivaudou

Last updated by author(s): 2023/11/28

Reporting Summary

Nature Portfolio wishes to improve the reproducibility of the work that we publish. This form provides structure for consistency and transparency in reporting. For further information on Nature Portfolio policies, see our Editorial Policies and the Editorial Policy Checklist.

Statistics

For all statistical analyses, confirm that the following items are present in the figure legend, table legend, main text, or Methods section.

n/a	Confirmed	
☐	☒	The exact sample size (*n*) for each experimental group/condition, given as a discrete number and unit of measurement
☐	☒	A statement on whether measurements were taken from distinct samples or whether the same sample was measured repeatedly
☐	☒	The statistical test(s) used AND whether they are one- or two-sided<br>*Only common tests should be described solely by name; describe more complex techniques in the Methods section.*
☐	☒	A description of all covariates tested
☐	☒	A description of any assumptions or corrections, such as tests of normality and adjustment for multiple comparisons
☐	☒	A full description of the statistical parameters including central tendency (e.g. means) or other basic estimates (e.g. regression coefficient) AND variation (e.g. standard deviation) or associated estimates of uncertainty (e.g. confidence intervals)
☐	☒	For null hypothesis testing, the test statistic (e.g. *F*, *t*, *r*) with confidence intervals, effect sizes, degrees of freedom and *P* value noted<br>*Give P values as exact values whenever suitable.*
☒	☐	For Bayesian analysis, information on the choice of priors and Markov chain Monte Carlo settings
☒	☐	For hierarchical and complex designs, identification of the appropriate level for tests and full reporting of outcomes
☒	☐	Estimates of effect sizes (e.g. Cohen's *d*, Pearson's *r*), indicating how they were calculated

Our web collection on statistics for biologists contains articles on many of the points above.

Software and code

Policy information about availability of computer code

Data collection	Commercial: pClamp 11.0.3, HiClamp software 1.2, MetaMorph 7.10.5.476, Zeiss Zen Black 2.3, Infinity Analyzer v7.1.0, Micro-Manager 1.4.21
Data analysis	Custom: eeFit 1.32 (published), eeTEVC 1.0 (published). Commercial: ImageJ 1.54 d, GraphPad Prism 8.0.1.

For manuscripts utilizing custom algorithms or software that are central to the research but not yet described in published literature, software must be made available to editors and reviewers. We strongly encourage code deposition in a community repository (e.g. GitHub). See the Nature Portfolio guidelines for submitting code & software for further information.

Data

Policy information about availability of data

All manuscripts must include a data availability statement. This statement should provide the following information, where applicable:

- Accession codes, unique identifiers, or web links for publicly available datasets
- A description of any restrictions on data availability
- For clinical datasets or third party data, please ensure that the statement adheres to our policy

The data that support the findings are provided in the Source Data file.

Research involving human participants, their data, or biological material

Policy information about studies with human participants or human data. See also policy information about sex, gender (identity/presentation), and sexual orientation and race, ethnicity and racism.

Reporting on sex and gender	N/A
Reporting on race, ethnicity, or other socially relevant groupings	N/A
Population characteristics	N/A
Recruitment	N/A
Ethics oversight	N/A

Note that full information on the approval of the study protocol must also be provided in the manuscript.

Field-specific reporting

Please select the one below that is the best fit for your research. If you are not sure, read the appropriate sections before making your selection.

☒ Life sciences ☐ Behavioural & social sciences ☐ Ecological, evolutionary & environmental sciences

For a reference copy of the document with all sections, see nature.com/documents/nr-reporting-summary-flat.pdf

Life sciences study design

All studies must disclose on these points even when the disclosure is negative.

Sample size	No statistical method was used to predetermine sample size. For each independent experiment sample size was maximized based on experimental logistic constraints (time, N max samples per run,N max samples per equipment). No technical replicates were used. All N indicated correspond to individual cells/oocytes/tadpoles except for luminescence measurements in Fig2a where each N corresponds to an average of 3 oocytes.
Data exclusions	No data was excluded except for maximal fluorescence changes and half time of fluorescence change where outliers were identified and removed using the ROUT (robust regression and outlier removal) method with a coefficient Q of 1%.
Replication	For each experiment, repetitions were performed until the inclusion of the last data batch would not change the variance of the total sample significantly. Experiments were repeated independently at least twice except for: the experiment in Fig5b which was performed on 5 independent tadpoles from 1 single batch; the experiments in Fig 1a, 1g (BAPTAin), Sup Fig 1 c (BAPTAin), Sup Fig 1 g,h, Sup Fig 6 (where n<7) and Sup Fig 9 b (ChR2, TARA150) that were performed in one single batch of oocytes; the experiments in Fig 3b, Sup Fig 5 that were performed in cells from one single transfection.
Randomization	Collected oocytes were selected at stage V-VI and randomly sorted into the different experimental conditions. Tadpoles were staged first and then randomly allocated to the different experimental conditions. Cells were transfected, identified for protein expression through the fluorescent marker of the transfected plasmid and then randomly allocated to the different experimental conditions. No scoring was used to distinguish and allocate individual members of either of the model cell/animals to the different experimental conditions.
Blinding	The investigators were not blinded to allocation during experiments and outcome assessment since all experiments were performed by single individuals in the absence of other staff members.

Reporting for specific materials, systems and methods

We require information from authors about some types of materials, experimental systems and methods used in many studies. Here, indicate whether each material, system or method listed is relevant to your study. If you are not sure if a list item applies to your research, read the appropriate section before selecting a response.

Materials & experimental systems

n/a	Involved in the study
☒	☐ Antibodies
☐	☒ Eukaryotic cell lines
☒	☐ Palaeontology and archaeology
☐	☒ Animals and other organisms
☒	☐ Clinical data
☒	☐ Dual use research of concern
☒	☐ Plants

Methods

n/a	Involved in the study
☒	☐ ChIP-seq
☒	☐ Flow cytometry
☒	☐ MRI-based neuroimaging

Eukaryotic cell lines

Policy information about cell lines and Sex and Gender in Research

Cell line source(s)	HEK293T obtained from ATCC #CRL-11268.
Authentication	Cells were originally authenticated by supplier, used regularly in recent years and not further authenticated.
Mycoplasma contamination	Cells were tested regularly and confirmed negative for mycoplasma contamination using the MycoAlert kit (Lonza).
Commonly misidentified lines (See ICLAC register)	None.

Animals and other research organisms

Policy information about studies involving animals; ARRIVE guidelines recommended for reporting animal research, and Sex and Gender in Research

Laboratory animals	Adult female Xenopus laevis (age not determined) for oocyte collection maintained for <1 year after reception from commercial supplier. Tadpoles for animal tests were used within 4 days of fertilization.
Wild animals	No wild animals were used in this study.
Reporting on sex	Xenopus laevis for oocyte collection were female. Tadpole sex was not identified.
Field-collected samples	No samples were field-collected.
Ethics oversight	Xenopus laevis handling and experiments fully conformed with European regulations and were approved by the Ethics Committee of the Commissariat à l'Energie Atomique et aux Energies Alternatives (Ethics Approval #12-040). Authorization of the animal facility has been delivered by the regional administration (Préfet de l'Isère, authorization # D 38 185 10 001).<br>Experiments on Xenopus tadpoles were carried out in accordance with the European Community Guide for Care and Use of Laboratory Animals and approved by the "Comité d'éthique en expérimentation de Bordeaux", N° 33011005-A.

Note that full information on the approval of the study protocol must also be provided in the manuscript.
