# Supplementary material for: Hijacking of internal calcium dynamics by intracellularly residing viral rhodopsins
Source: Nat Commun. 2024 Jan 2;15:65. doi: 10.1038/s41467-023-44548-6 (PMC10761956; doi:10.1038/s41467-023-44548-6)
Supplement: Supplementary file 2 — Peer Review File [file 41467_2023_44548_MOESM2_ESM.pdf]

nature portfolio

Peer Review File

Hijacking of internal calcium dynamics by intracellularly residing viral rhodopsins

Open Access This file is licensed under a Creative Commons Attribution 4.0 International License, which permits use, sharing, adaptation, distribution and reproduction in any medium or format, as long as you give appropriate credit to the original author(s) and the source, provide a link to the Creative Commons license, and indicate if changes were made. In the cases where the authors are anonymous, such as is the case for the reports of anonymous peer reviewers, author attribution should be to 'Anonymous Referee' followed by a clear attribution to the source work. The images or other third party material in this file are included in the article's Creative Commons license, unless indicated otherwise in a credit line to the material. If material is not included in the article's Creative Commons license and your intended use is not permitted by statutory regulation or exceeds the permitted use, you will need to obtain permission directly from the copyright holder. To view a copy of this license, visit http://creativecommons.org/licenses/by/4.0/.

REVIEWER COMMENTS

Reviewer #1 (Remarks to the Author):

Viral rhodopsins are a new group of microbial rhodopsins. Most of them show ion channel functions in a light-dependent manner. In this work, Eria-Oliveira et al. found OLPVR1 and VCR1s, members of viral rhodopsins, are specifically located in ER in Xenopus oocytes and mammalian cells and induce CaCC through a calcium release stronger than that can be achieved by other ion-transporting microbial rhodopsins from ER. Their findings would not only pave the way for the development of new optogenetic tools that can control intracellular calcium levels without affecting the membrane potential of the cells but also suggest a new physiological role of viral rhodopsins played in the host algae cells in nature. Their experiments were well organized, and the results sufficiently support their conclusions. I recommend this article be published in Nature Communications but a few minor points that should be addressed by the authors before the publication are listed below.

Line 114-115 “In fact, CaCC response after OLPVR1 illumination was twice as slow as that after M3 activation by ACh”

Since M3 activation induces calcium release through multi-step signaling, it could take more time compared to the direct release of ER calcium by OLPVR1. Please describe possible reasons for the larger latency after the OLPVR1 illumination than that of the M3 activation.

Line 123-124 “Analysis of the atomic structure of OLPVR1 and its high phylogenetic similarity to VirChR1 (61% sequence identity)”

It would be useful to include a full alignment of amino acid sequences (not the list of sequences) of viral rhodopsins studied in this work in the Supplementary Information.

Fig. 2F

The green area indicating illumination appears to be displaced with respect to the actual time period of the illumination. Please check it.

Line 210

“BAPTA-AM” appeared for the first time here in the manuscript. Please indicate the difference in its meaning from that in “BAPTA”.

Line 293

"data not shown" does not much with the guidelines of Nat. Commun. (https://www.nature.com/ncomms/submit/how-to-submit). I recommend adding corresponding experimental results in the Supplementary Information.

Movie S2

They used red-light flushes in this experiment. It is different from continuous illuminations of green-light experiments in other movies. I could not find an explanation of the reason why the authors used different light exposure patterns between red and green light in the manuscript. Please add an explanation of this issue and, if it is possible, a result of a new experiment with continuous red-light illumination.

Reviewer #2 (Remarks to the Author):

The manuscript by Ana-Sofia Eria-Oliveira et al. describes molecular properties and optogenetics application of viral channelrhodopsins (VCR1s), mainly OLPVR1.

The authors previously reported the molecular structure of OLPVR1 and the light-driven proton transport (Dmitrii Zabelskii et al Nat. Commun (2020) 11:5707). They also demonstrated the homologous protein VirChR1 is a light-gated cation channel that transports Na+ and K+. Interestingly, the channel is blocked by Ca2+.

Here, the authors showed that VCR1s are localized intracellularly (mainly on the ER membrane) when heterologously expressed in Oocyte or HEK293T cells and that it did induce elevation of the cytoplasmic Ca2+ level, resulting in the activation of Ca-gated ion channels. The authors successfully demonstrated optogenetics control of the behavior of Xenopus tadpoles by using OLPVR1.

It is obvious that OLPVR1-expressing cells exhibit light-dependent Ca-dynamics change, and VCR1s would be novel optogenetics tools for modulating biological behaviors without altering the electric potential of the plasma membrane. Thus, the work is original and of large significance in rhodopsin research and neuroscience research.

However, it is ambiguous whether the OLPVR1 directly conducts Ca2+ or other ions, or induces activation of some Ca2+ channels (such as IP3 receptor) located in ER.

Because of the poor localization of OLPVR1 on the plasma membrane, the authors attempted the number of variants for improving the expression (Fig. S6), which unfortunately did not work. Therefore, they were not able to directly demonstrate the OPLVR1 photocurrent by patch-clamp.

Furthermore, the authors previously reported that the ion channel activity of the VirChR1 (homolog of OLPVR1) is blocked by Ca2+ (Kd=2.2 mM). Although the cytoplasmic Ca2+ level is by far low (as the

authors argued in the discussion part), the local Ca2+ level close vicinity of the ER could be elevated upon Ca2+ release from the ER. Actually, they observed an increase in the Ca2+ signal (Fig4B, traces 1, 3 and 4).

Thus, when the OLPVR1 is located in ER, it is not convincing that the OLPVR1 transports Ca2+ as depicted in Fig. 4A.

Other points

Lines 250-252

“To isolate events of muscle contraction we subjected a subset of OLPVR1-injected light-responsive tadpoles to the neuromuscular junction blocker tubocurarine, as well as MS-222/tricaine, an anesthetic blocking sensory-motor transmission.”

Please add the effect of each blocker and explain Fig 5D and E in more detail.

Lines 281-284

“If animal rhodopsins natively reside in internal membranes, it is accepted that microbial channelrhodopsins, such as Chlamydomonas reinhardtii ChR, natively localize and function at the surface membrane. Our results challenge this view by demonstrating that channelrhodopsins can have a biological function in internal membranes.”

It has been previously shown that Chlamydomonas reinhardtii ChR is located in the eye-spot, not at the surface membrane in the native cell (Berthold et al. The Plant Cell, 20, 1665–1677 2008). Please correct the sentences.

Reviewer #3 (Remarks to the Author):

This is an interesting paper.

The same group reported the structure of OLPVR1 three years ago (Zabelskii et al. Nat. Commun. 2020). In the paper, the authors suggested channel function for OLPVR1, but they failed measuring ion-transport of OLPVR1 by light. Light-gated channel functions were measured for other viral rhodopsins. Interestingly, the structure of the active center in OLPVR1 resembles that of a light-driven proton-pump bacteriorhodopsin. Thus, it was an interesting paper, but the story was so complicated.

In this paper, the authors found expression of OLPVR1 to the endoplasmic reticulum in Xenopus oocytes and a mammalian cell line, and an increase in cytoplasmic calcium by light. These results provide a new function of rhodopsin, and useful in optogenetics. In fact, in this paper, they demonstrated muscle contraction by light. I agree with the authors; these rhodopsins can be novel optogenetics tools that release calcium without altering plasma membrane electrical properties. The experiments were thoroughly performed, and their interpretation is reasonable.

Only one regrettable thing is that we still do not know the activation mechanism of OLPVR1. They suggested calcium channel function for OLPVR1, but without direct evidence. In Supplementary Figure S6, they tested various constructs, but without successful surface expression.

In conclusion, as the information is novel, I recommend publication of this manuscript. Fig. 2F should be properly changed (illumination time must be wrong).

Point-by-point reply to the reviewers

We thank the reviewers for their time and effort in evaluating our work, and the useful analysis, critics, and suggestions for improvement, of our data and conclusions.

Reviewers' verbatim remarks are in blue bold, and our answers in normal text. Changes to the manuscript text are highlighted in yellow, here and in the manuscript.

Reviewer #1 (Remarks to the Author):

Line 114-115 "In fact, CaCC response after OLPVR1 illumination was twice as slow as that after M3 activation by ACh"

Since M3 activation induces calcium release through multi-step signaling, it could take more time compared to the direct release of ER calcium by OLPVR1. Please describe possible reasons for the larger latency after the OLPVR1 illumination than that of the M3 activation.

While the Ca^{2+} release upon M3 activation is carried solely by IP_3R (IP_3 Receptors), the same is not true upon light-activation of OLPVR1. A possible explanation is that the slower activation of TMEM16A currents by OLPVR1 could be a consequence of a smaller Ca^{2+} conductance of OLPVR1 channels compared to IP_3R. Indeed, assuming OLPVR1 has a conductance similar to ChR2 (~100 fS [Feldbauer et al, PNAS, 2009], the OLPVR1 conductance would be ~1000-fold smaller than that of IP_3R (~50-300 pS [Foskett et al, Physiol Rev, 2007]).

A much more direct explanation arises from the different extents of activation of M3 and OLPVR1 as a consequence of the distinct stimuli applied. As we discuss on page 6 of the manuscript, bath-applied ACh reaches the entire population of surface M3 receptors and the produced IP_3 molecules can diffuse to reach most intracellular IP_3R. In contrast, light can only penetrate a few microns below the surface of opaque oocytes and reaches a small fraction of the OLPVR1 proteins present. This means the global extent of Ca^{2+} release is much higher for M3-expressing cells compared to OLPVR1-expressing cells. Because the increase in cytoplasmic Ca^{2+} is higher in M3-expressing cells, the concentration gradient between the ER vicinity and the surface membrane - where TMEM16A channels are located – is more pronounced. According to Fick's first law of diffusion, the rate of diffusion is therefore expected to be faster, which is reflected as a faster activation of TMEM16A currents. This difference in both amplitude and rate of activation of TMEM16A currents is further exacerbated by the fact that there is a positive cooperative Ca^{2+} activation of TMEM16A.

To address this concern, we now write in the revised manuscript (page 6):

"We also note that M3 stimulation produced ~20-fold larger and ~2-fold faster currents than OLPVR1. Such difference is not unexpected considering the different natures of the stimuli. Bath-applied ACh reaches the entire population of surface M3 receptors and the produced IP_3 molecules can diffuse to reach most intracellular IP_3 receptors. In contrast, light can only penetrate a few microns below the surface of opaque oocytes and reaches a small fraction (<5%) of the OLPVR1 proteins present (See Methods). As such, the extent of Ca^{2+} released from the ER upon M3 activation is much higher and the Ca^{2+} concentration near the surface membrane where CaCCs reside reaches the CaCC activation threshold faster."

Line 123-124 "Analysis of the atomic structure of OLPVR1 and its high phylogenetic similarity to VirChR1 (61% sequence identity)"

It would be useful to include a full alignment of amino acid sequences (not the list of sequences) of viral rhodopsins studied in this work in the Supplementary Information.

We agree with the reviewer. An alignment of VCR1 rhodopsins is now included in the supplementary information of the manuscript.

Fig. 2F

The green area indicating illumination appears to be displaced with respect to the actual time period of the illumination. Please check it.

The green area was inadvertently moved. We thank the reviewer for noticing the mistake. Fig. 2F has been corrected in the revised manuscript.

Line 210

“BAPTA-AM” appeared for the first time here in the manuscript. Please indicate the difference in its meaning from that in “BAPTA”.

We address now this point on page 9 of the revised manuscript:

“Incubating the cells with 15 µM BAPTA-AM - a membrane permeant inactive BAPTA derivative that is converted to the membrane impermeant active BAPTA in the cell cytoplasm - for 2h was sufficient to chelate bulk cytosolic Ca^{2+} as reported by the absence of signal from free cytosolic GCaMP6s (Fig. 4, conditions 1&2).”

Line 293

“data not shown” does not much with the guidelines of Nat. Commun. (https://www.nature.com/ncomms/submit/how-to-submit). I recommend adding corresponding experimental results in the Supplementary Information.

We agree with the reviewer. To replace the “data not shown” statement, we have added an additional supplementary figure (Supplementary Figure S10) where we show:

(A) Confocal images of $ChR2_{ER}$-expressing HEK293T cells;

(B) Average maximal fluorescence changes of cells transfected with ER voltage sensor $ASAP3_{ER}$ alone or with $ChR2_{ER}$ upon illumination.

(C) Electrophysiological traces of HEK293T cells co-transfected with $ChR2_{ER}$ as well as the calcium-activated chloride channel TMEM16A under illumination;

(D) the average light-triggered current increase of the different cells tested.

Movie S2

They used red-light flushes in this experiment. It is different from continuous illuminations of green-light experiments in other movies. I could not find an explanation of the reason why the authors used different light exposure patterns between red and green light in the manuscript. Please add an explanation of this issue and, if it is possible, a result of a new experiment with continuous red-light illumination.

Both green and red illuminations on the supplementary videos were in fact performed under continuous light application using an Alonefire X004 lamp that emits both continuous green (510-530 nm) or red light (600-720 nm). We recognise that, in the red-light illumination video S2, continuous light does appear as flashes. However, that is only an optical illusion, a consequence of the longer wavelength of red light in combination with the camera and video

acquisition sampling settings, akin to the ‘wagon-wheel’ stroboscopic effect. To clarify this issue, we added this same explanation to the description of the supplementary videos.

Reviewer #2 (Remarks to the Author):

Furthermore, the authors previously reported that the ion channel activity of the VirChR1 (homolog of OLPVR1) is blocked by Ca^{2+} (Kd=2.2 mM). Although the cytoplasmic Ca^{2+} level is by far low (as the authors argued in the discussion part), the local Ca^{2+} level close vicinity of the ER could be elevated upon Ca^{2+} release from the ER. Actually, they observed an increase in the Ca^{2+} signal (Fig4B, traces 1, 3 and 4). Thus, when the OLPVR1 is located in ER, it is not convincing that the OLPVR1 transports Ca^{2+} as depicted in Fig. 4A.

Indeed, in Zabelskii et al [Nat Comm, 2020] it is demonstrated that VirChR1 is blocked by Ca^{2+} (Kd=2.2 mM). However, in these experiments the Ca^{2+} was in fact applied extracellularly, thus bathing the extracellular face of the protein. The extracellular side of the protein is presumed to face the lumen of the ER rather than the cytosol, as it is the case with transmembrane proteins that are translocated at the ER membrane and sorted to the plasma membrane during protein maturation. The lumen of the ER does not exceed hundreds of µM Ca^{2+} (Ref 29). In Fig 3J of Zabelskii et al [2020], we can see that only at >1mM does Ca^{2+} have a significant effect on VirChR1 photocurrents. Thus, it is not expected that the ER Ca^{2+} concentration will block OLPVR1 activity.

We agree that local Ca^{2+} concentrations around microdomains of Ca^{2+} permeation reach much higher values than the global Ca^{2+} cytosolic concentration (~100 nM). Illuminating OLPVR1 would trigger Ca^{2+} permeation from the ER to the cytosol, following its concentration gradient. In this situation the higher local concentrations of Ca^{2+} would bathe the cytosolic face of the protein, rather than the extracellular face. We have no indication that Ca^{2+} blocks through the intracellular cytosolic face of the protein. Regardless, even if that were the case, local microdomain Ca^{2+} concentrations are neither estimated nor described to reach inhibitory mM range [Parekh, J Physiol, 2008].

Taking that into consideration, the increase in Ca^{2+} signal seen in Fig 4B (traces 1, 3 and 4) is expected.

It is quite fascinating that this block by Ca^{2+} happens only at concentrations that do not exist intracellularly but are found in the high saline extracellular environment of the native hosts of the viral rhodopsins. This ensures that any viral rhodopsin that might fortuitously reach the plasma membrane is fully blocked by high ambient Ca^{2+}. Together with the observation that VCR1s have a strong predisposition to reside in intracellular membranes, this reiterates how their localization seems to be key for their proper function and role in virus-host interactions.

Furthermore, even though we did not measure direct Ca^{2+} currents through OLPVR1, we do have two independent pieces of evidence that together strongly indicate direct OLPVR1 calcium permeation.

The first, in oocytes, is seen in Fig 2G-H, where we show there is a fraction of calcium release that is IP_3R-independent, since it is not blocked by 2-APB. As Xenopus oocytes do not express other calcium release channels [Kobrinsky et al, Dev Biol, 1995], the remaining fraction is likely carried by OLPVR1.

The second piece of evidence, now in HEK293T cells, is seen in Fig 4B (trace 4) and C, together with Supplementary Figure S8.

In Supplementary Figure 8 we demonstrate that BAPTA is sufficient to block Ca^{2+} permeating through neighbouring proteins to reach the Ca^{2+} sensor attached to loss-of function OLPVR1(K204Q). To elaborate further, when wild-type OLPVR1 is co-expressed with OLPVR1(K204Q)-GCaMP6S, upon illumination, an increase in cell fluorescence is observed. The increase of fluorescence is the consequence of an increase of Ca^{2+} in the proximity of the

fluorescent Ca^{2+} sensor GCaMP6S. GCaMP6s is fused to OLPVR1(K204Q), which is a loss-of-function mutant that itself does not permeate Ca^{2+} . This means the increase in Ca^{2+} around OLPVR1(K204Q)-GCaMP6S is arising from Ca^{2+} permeation from neighbouring proteins, i.e., co-expressed wild-type OLPVR1 proteins or native IP_3R. In the same conditions but with BAPTA in the cytoplasm, light activation of OLPVR1 does not produce an increase in fluorescence. This means that BAPTA is sufficient to chelate Ca^{2+} ions entering from the SR through neighbouring wild-type OLPVR1 proteins or native IP_3R before they reach OLPVR1(K204Q)-GCaMP6S. The significance of this experiment is that it establishes that BAPTA shields the Ca^{2+} sensor GCaMP6S from any Ca^{2+} ions originating from surrounding proteins, but not from the protein fused to the GCaMP6s sensor, i.e., OLPVR1 in Fig 4.

In Fig 4B trace 4, in the presence of BAPTA, we still observe an increase in the Ca^{2+} fluorescence signal in cells expressing OLPVR1-GCaMP6s. Thus, taking into consideration the control experiments of Supplementary Figure S8, this remaining Ca^{2+} signal in the presence of BAPTA cannot be explained by Ca^{2+} influx through neighboring proteins. This BAPTA-insensitive signal must originate within the confines of OLPVR1-GCaMP6S, supporting a direct permeation of Ca^{2+} through OLPVR1.

In conclusion, while we cannot deny that the optimal experiment would be to directly record OLPVR1 currents, the evidence supports a direct permeation of calcium.

Other points

Lines 250-252

“To isolate events of muscle contraction we subjected a subset of OLPVR1-injected light-responsive tadpoles to the neuromuscular junction blocker tubocurarine, as well as MS-222/tricaine, an anesthetic blocking sensory-motor transmission.”

Please add the effect of each blocker and explain Fig 5D and E in more detail.

Please add the effect of each blocker…

We are not sure whether the reviewer is referring to adding further explanation of the known mechanism of action of each blocker or to add in Fig 5B the independent effect of each blocker on the tadpole light-response. We are then going to address both aspects.

Tubocurarine acts as an antagonist of nicotinic acetylcholine receptors in muscle cells at the neuro-muscular junction. Thus, tubocurarine blocks synaptic transmission between neurons and myocytes, acting as a paralytic drug [Wenningmann et al, Mol Pharmacol, 2002]. MS-222, or tricaine, is a sulfonated analogue of benzocaine that has been shown to effectively block the generation of action potentials through reversible interactions with voltage-dependent Na^+-channels in excitable cells, such as neurons and muscle cells. While tubocurarine acts as a pure paralytic, MS-222 acts as an analgesic and sedative as well, quenching pain and sensory responses [Ramlochansingh et al, PLoS One, 2014].

Since our ultimate goal was to isolate muscle contraction initiated from sarcoplasmic reticulum (SR) calcium release, we designed the experiments focused on that goal alone and we did not perform the experiments on OLPVR1-expressing tadpoles with each blocker independently. A combination of both blockers is certainly an overkill but, given the complexity of whole animal models, it reduces the possibility of ambiguous results and misinterpretation because we block the two muscle contraction triggers upstream of SR calcium release (i.e., nicotinic receptors and voltage-dependent Na^+-channels).

In non-muscle cells, testing each blocker alone might allow to dissect further the behavioural result of expression and activation of OLPVR1, for instance in motor neurons or sensory organs. There are already several characterized optogenetic tools for that effect. We find that

the competitive advantage of OLPVR1 is its calcium-release function. Muscle contraction is the most straightforward way to evaluate the usefulness of OLPVR1 as an intracellular calcium-release optogenetic tool and thus our decision of isolating muscle contraction by applying both blockers.

While we did not test the effect of each blocker on OLPVR1-expressing tadpoles, we did however test each blocker independently on control (non-injected) tadpoles to determine the effective concentrations of each compound. The concentration of each compound was chosen as the lowest concentration at which tadpoles loose muscle tone and response to mechanical stimulation. This ensured effectiveness of each compound with minimal non-specific effects arising from excessive doses. Below is a figure of these control experiments. We did not include this figure in the supplementary data because we did not consider it of sufficient merit

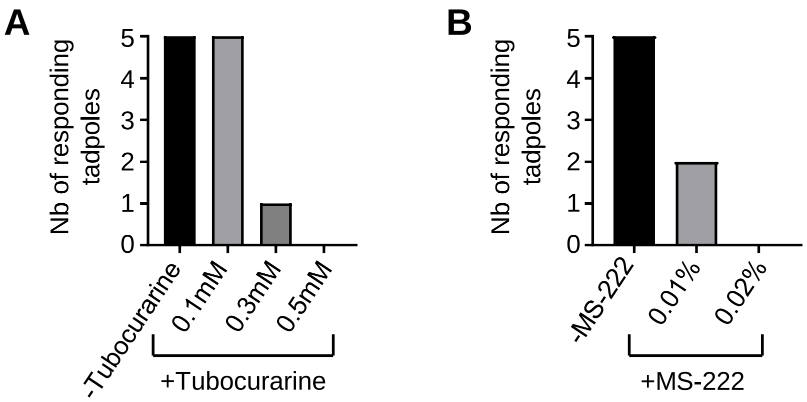

since we found the same active concentrations as most of the previously published works.

Auxiliary Fig. 1. Determination of the effective concentrations of tubocurarine and MS-222 in *Xenopus* tadpoles. Non-injected *Xenopus* tadpoles were subjected to different concentrations of either tubocurarine or MS-222 for 20 min and then observed for mechanosensitive response. For that purpose, each tadpole was exposed to a rapid flow of extracellular physiological solution directed at the posterior section of the body three consecutive times. A tadpole was accounted as responsive if it moved at least once in response to the three stimuli. (A) Number of tadpoles responding to mechanical stimuli under the effect of different concentrations of tubocurarine. (B) *Idem* for MS-222. At 0.5 mM of tubocurarine or 0.02% of MS-222 no tadpole was responsive to mechanical stimuli.

… explain Fig 5D and E in more detail.

As requested, we developed on the data shown in Fig 5D and E on page 11 of the revised manuscript:

"In Fig. 5 D we summarize in a Venn diagram the different behavioural responses observed in a series of 40 consecutive illuminations on a single tadpole. The responses to light were a combination of tail flicking, swimming and twitching. Tail flicking was the most frequent behaviour appearing in 83% of illumination instances. We further examined the time onset and offset of the observed behaviours (Fig. 5 E). Tail flicking started and ended within 2 s when light was switched on and off. Swimming started more slowly, within 6 s, and stopped after >15 s. The time frame of tail flicking is consistent with OLPVR1 light-induced Ca2+ release and muscle contraction. Since OLPVR1 expression was not targeted to any particular cell type, effects of OLPVR1-activation in different tissues other than muscle were not

excluded in these experiments. To isolate events of muscle contraction we subjected a subset of OLPVR1-injected light-responsive tadpoles to the neuromuscular junction blocker tubocurarine, as well as MS-222/tricaine, an anesthetic blocking sensory-motor transmission. Four out of the five tadpoles tested retained light responsiveness (Fig. 5 B) in the form of tail flicking, shown by the single behaviour observed upon 40 consecutive illuminations (Fig. 5 E). These experiments suggest that tail flicking, but not swimming, is a direct result of triggering OLPVR1-mediated Ca2+ release in the muscle cells of the tadpoles.“

Lines 281-284

“If animal rhodopsins natively reside in internal membranes, it is accepted that microbial channelrhodopsins, such as Chlamydomonas reinhardtii ChR, natively localize and function at the surface membrane. Our results challenge this view by demonstrating that channelrhodopsins can have a biological function in internal membranes.”

It has been previously shown that Chlamydomonas reinhardtii ChR is located in the eye-spot, not at the surface membrane in the native cell (Berthold et al. The Plant Cell, 20, 1665–1677 2008). Please correct the sentences.

Indeed, Berhold et al [The Plant Cell, 2008] showed that ChR is located in the eyespot. However, the eyespot structure is a specialized photoreceptive apparatus in part composed of plasma membrane. A complete integrated schematics of the eyespot structure can be found in Fig 1 of Seth et al [*The structure and functional mechanism of eyespot in Chlamydomonas*. J Basic Microbiol, 2022, 62:1169-1178].

Interestingly it is also in Berhold et al [The Plant Cell, 2008] that we can find the demonstration that ChR resides in the plasma membrane fraction of the eyespot, though not explicitly stated by the authors. Their recordings of ChR currents involve patch clamping techniques in a configuration that they call “eyespot in”, which is a variation of cell-attached patch clamp. In this configuration, they use cell wall-deficient intact C. reinhardtii cells, and they use a patch pipette to create a seal at the plasma membrane. The particularity of their patch configuration is that they patch the plasma membrane on the eyespot region, easily identified by the orange coloration. This patch configuration allows to record ionic currents through the patch of membrane sealed to the pipette. Thus, the photocurrents they observed are in fact originating from ChR at the plasma membrane of the cell, directly above the chloroplast envelopes and layers of carotenoid pigment granules of the eyespot.

We recognise that our statement might be misleading, because indeed ChR are not expressed uniformly at the plasma membrane, and thus we modified our sentence on page 13 of the revised manuscript:

“If animal rhodopsins natively reside in internal membranes, it is accepted that microbial channelrhodopsins, such as Chlamydomonas reinhardtii ChR2 , natively localize and function at the surface membrane, characteristically in the plasma membrane fraction of specialized eyespot structures.“

Reviewer #3 (Remarks to the Author):

Only one regrettable thing is that we still do not know the activation mechanism of OLPVR1. They suggested calcium channel function for OLPVR1, but without direct evidence. In Supplementary Figure S6, they tested various constructs, but without successful surface expression.

Indeed, despite many attempts we were unsuccessful in achieving sufficient surface expression for electrophysiological characterization. We should add that, in earlier work during structural characterization, there were attempts to reconstitute the purified protein in black-lipid membranes for functional characterization, which proved to be unsuccessful as well.

Since OLPVR1 is a short protein (223 aminoacids), with no identifiable signal sequence, one could question what, in fact, retains the protein at the ER. We suspect that lipid context and interaction might be at the source of this strong predisposition. In Zabelskii et al [Nat Comm, 2020], OLPVR1 structure shows a lipid fragment bound within the permeation pathway of the protein. A mutated version of OLPVR1 that excluded the lipid binding, the O1O2 mutant was also structurally characterized [Supplementary information of Zabelskii et al, Nat Comm, 2020] and we found that it expressed at similar levels as wild-type OLPVR1 but was electrophysiologically silent. Understanding this predisposition for internal localization will be fundamental for the engineering of the protein for direct characterization.

Instead, here we rather focused and combined evidence on the mobilization of intracellular calcium by OLPVR1.

Despite the lack of direct recordings of OLPVR1 current we do present two independent pieces of evidence to support direct calcium permeation through OLPVR1:

- The first, in oocytes, is presented in Fig 2G-H, where we show that a fraction of calcium release is IP_3R-independent, which, considering the lack of other calcium release channels in Xenopus [Kobrinsky et al, Dev Biol, 1995], is likely carried directly by OLPVR1;

- The second piece of evidence, in HEK293T cells, is seen in Fig 4 B (trace 4) and C. There, we demonstrate fluorescence increase in cells expressing OLPVR1-GCaMP6s, even in chelating BAPTA-conditions which, see Supplementary Figure S8, are sufficient to remove the contribution of calcium increase from surrounding proteins. Thus, the recorded fluorescence is likely a consequence of direct Ca^{2+} permeation through GCaMP6s-fused OLPVR1.

Together, both results strongly suggest direct calcium permeation by OLPVR1.

Fig. 2F should be properly changed (illumination time must be wrong).

The green area was indeed incorrectly placed. We thank the reviewer for noticing the mistake. Fig. 2F has been corrected in the revised manuscript.

REVIEWERS' COMMENTS

Reviewer #1 (Remarks to the Author):

The authors appropriately addressed to the reviewer's comments and revised the description in their manuscript, which made their work suitable for publication in Nat. Commun. This reviewer recommends accepting for the publication.

Reviewer #2 (Remarks to the Author):

I thank the authors for answering all my questions and comments. As the authors explained, various experiments with Oocytes and HEK293 cells support calcium conductance through OLPVR1. And it is very interesting that OLPVR1 conducts Ca2+, but the activity is blocked by a high Ca2+. Hope the significance of this phenomenon will be revealed in the future.

Point-by-point reply to the reviewers

We thank the reviewers for their positive comments.

Reviewers' verbatim remarks, below, do not specify any further issue requiring an answer.

Reviewer #1 (Remarks to the Author):

The authors appropriately addressed to the reviewer's comments and revised the description in their manuscript, which made their work suitable for publication in Nat. Commun. This reviewer recommends accepting for the publication.

Reviewer #2 (Remarks to the Author):

I thank the authors for answering all my questions and comments. As the authors explained, various experiments with Oocytes and HEK293 cells support calcium conductance through OLPVR1. And it is very interesting that OLPVR1 conducts Ca2+, but the activity is blocked by a high Ca2+. Hope the significance of this phenomenon will be revealed in the future.
